# The effectiveness of non-pharmacological interventions for low back pain in China: A systematic review and network meta-analysis

**DOI:** 10.1371/journal.pone.0322929

**Published:** 2025-05-09

**Authors:** Junshi Liu, Weng Kung Peng, Yingxian Jia

**Affiliations:** 1 Department of Physical Education, Education School, Dongguan University of Technology, Dongguan City, Guangdong Province, China; 2 Graduate School of Engineering Science, Osaka University, Osaka, Japan; 3 Department of Reproductive Medicine, Guizhou Provincial People’s Hospital, Guiyang City, Guizhou Province, China; Korea University Medical Center, KOREA, REPUBLIC OF

## Abstract

Low back pain (LBP) is the most common and incapacitating musculoskeletal disorder globally. The use of non-pharmacological interventions for the management of LBP is recommended by global clinical practice guidelines. However, existing guidelines often exhibit poor quality, as evidenced by inadequate systematic reviews of evidence. This necessitates further reviews tailored to the specific context of different countries. This review searched the CINAHL, EMBASE, MEDLINE, The Cochrane Library, Web of Science, and Wanfang databases from their inception to May 14, 2024. After data extraction by two independent researchers, a total of 57 randomized control trials (RCTs) were included. The Bayesian network meta-analysis results demonstrated that: (1) non-pharmacological therapies generally exhibited superior efficacy over pharmaceuticals in improving functional disability and overall efficacy; (2) pharmaceuticals, both alone and in combination with non-pharmacological therapies, were generally more effective than most non-pharmacological therapies in reducing pain intensity. High heterogeneity was observed, which could be explained by LBP subtypes in the analysis of functional disability. While heterogeneity had a limited impact on the confidence of results for pain intensity and functional disability, it significantly influenced the assessment of overall efficacy with major concerns of imprecision. The high volume of studies with a high risk of overall bias necessitates cautious interpretation of these findings. Chinese LBP patients may benefit most from non-pharmacological interventions, particularly those rooted in Traditional Chinese Medicine, for improving disability. For pain intensity reduction, pharmaceuticals and multi-component therapies incorporating pharmaceuticals may be more effective.

## Introduction

Low back pain (LBP) is a well-known disabling musculoskeletal disorder with the highest prevalence worldwide [1]. The clinical course of LBP can be divided into an acute phase (< 6 weeks), a subacute phase (6 weeks to 12 weeks), and a chronic phase (> 12 weeks) [2]. The nociceptive input model identifies three primary causes of LBP: an unidentifiable source (non-specific LBP), a neurological source, and a specific pathology [3]. LBP with a specific cause accounted for a small proportion, with non-specific LBP reported most frequently. While clinical guidelines for LBP management have been established, a significant gap persists globally between evidence-based recommendations and actual clinical practice [2]. Despite income disparities, intervention guidelines, often derived from research in wealthier nations, are frequently applied universally. Methods developed in these countries may face issues such as cultural acceptance, patient and provider attitudes toward and compliance with these methods, and health-care system capacity in low-income countries. Despite the challenges in developing consistent clinical guidelines for LBP management, there is growing support for the use of non-pharmacological interventions, as evidenced by increasing recommendations [4]. However, assessments of existing guidelines have revealed significant quality issues. Inadequate reporting of barriers to implementation, limited information on implementation tools, and a lack of comprehensive systematic evidence reviews may hinder the effectiveness of these guidelines. For the global LBP management, it is therefore essential to enhance these domains and keep creating evidence for guidelines establishment in accordance with the requirements and capabilities of the relevant countries.

China faces an accelerated aging population, resulting in a high prevalence of LBP patients with significant disability-adjusted life years [5]. It is recommended that LBP be managed using a combination of pharmacological and non-pharmacological methods [6–8]. Furthermore, many clinical guidelines prioritized pharmaceuticals as the first-line intervention option [8]. Major clinical guidelines for non-pharmacological interventions did not align with the recommendation of prioritizing pharmaceuticals [9]. In addition, existing guidelines provided weak recommendations for the use of non-pharmacological interventions [6]. In the clinical practice guidelines assessment, non-pharmacological intervention like acupuncture received low ratings [7]. Currently, there is a dearth of SR-MA (systematic reviews and meta-analyses) studies in China specifically examining the effectiveness of non-pharmacological interventions for LBP. Therefore, the development of these guidelines was dependent on the advice of experts and evidence that was out of date. Our assumptions are supported by ongoing research, which primarily focuses on chronic pain in older adults and has been published as protocols or limited systematic reviews [10]. SR-MA studies should focus on key domains, including: various characteristics of LBP, specifics of intervention implementation, demographic characteristics of Chinese patients, treatment effectiveness (both pre- and post-intervention), factors that can enhance guideline appraisal and evaluation. These findings will be crucial for developing high-quality clinical practice guidelines on non-pharmacological interventions for LBP in China [4].

This study aimed to evaluate the impact of existing non-pharmacological intervention trials for adults with LBP in China through the application of systematic review and network meta-analysis (NMA). Recognizing the limitations of direct evidence comparing non-pharmacological interventions and pharmaceuticals, a NMA was deemed feasible as it could utilize indirect evidence to make these comparisons. Our goal was to present the most recent evidence on the characteristics of trials and the effectiveness of comparisons between these interventions.

## Materials and Methods

### Search strategies

The Cochrane Library, MEDLINE, EMBASE, CINAHL, Web of Science and Wanfang electronic databases were utilized from the time of their creation until May 14th, 2024. Using the Boolean operators “AND” and “OR”, search terms were connected (S1 Table). The Mendeley platform (version 1.19.8) was updated with the identified articles and duplicates were eliminated. Titles and abstracts of the identified articles were screened by two independent, blinded researchers. Disagreements about eligibility were resolved by consulting a third researcher who reviewed the relevant information and, if necessary, the full text. This study was registered in PROSPERO with CRD42024578680. Study protocol was provided in the file of supporting information (S2 Protocol).

### Eligibility criteria

The eligibility criteria for the included papers were set up using the PICO format. The following criteria were used for inclusion: (1) subjects with LBP subtypes over the age of 18 who received non-pharmacological intervention in the experimental group of randomized controlled trials; (2) quantitative data on the intervention’s effectiveness was present in the outcome measures; (3) the studies were conducted in China, with the Chinese residents. The exclusion criteria were as follows: (1) studies published as protocols, trial registry records, pilot trials, crossover trials, reviews, abstracts, comments, brief communications, discussions, theses, conference proceedings, and retracted articles; (2) trials with healthy subjects in the control group without any risk-minimization strategy; (3) trials only had experimental groups using intervention without a comparator having no intervention, sham intervention, minimal intervention, alternative intervention, or usual care; (4) the intervention targeted an underlying disease characterized by symptoms of LBP as a minor problem; (5) nerve blocks were used in the intervention; (6) LBP resulted from surgery or postpartum pain; (7) LBP occurred during labor in women; (8) the experimental group experienced aches from other parts of the body, like chest or shoulder pain; (9) the evaluation of the intervention’s effectiveness was limited to nursing or pain management. Information was subsequently extracted from full-text English-written eligible articles.

### Information extraction and data synthesis

Information extracted included: authors, publication year of the articles, patient demographics (age and gender), LBP characteristics (e.g., subtypes), intervention details (e.g., group size, number of sessions, session frequency, intervention duration), outcome measurement of the quantitative effectiveness of the intervention, and assessment timepoint.

The quantitative outcomes (i.e., mean, standard deviations, sample size, and positive events) of the effectiveness of the intervention with reference to pain intensity, functional disability, overall efficacy, quality of life, mental health, physical performance, and overall health at the pre- and post-intervention timepoint were chosen for data extraction. When multiple tools were available to assess the effectiveness of an intervention within a trial, priority was given to the tool that used the same scale as most of the other trials included in the analysis. If different measurement tools were used for the same outcome, the direction of the scales was first standardized by subtracting the mean from the maximum value. Subsequently, if the scales differed in magnitude, pain intensity scores were rescaled to a 0–10 range (0 indicates no pain), while functional disability scores were rescaled to a 0–100 range (0 indicates sever dysfunction). It is common and accepted practice to rescale data when assessing the effectiveness of treatments for LBP [11]. Results were taken from the tool that indicated the most ineffective cases if more than two tools were used to assess the overall efficacy.

The most recent visit to the post-intervention was taken into consideration in trials where outcome measurement was delayed after the end of intervention. When there was no direct report of the mean and standard deviations in the post-intervention period, Chi et al.‘s recommendation was followed to impute these numbers, which suggests calculating the numbers using extracted results such as the median, interquartile range, confidence interval, and normal distributions [12]. To address multi-arm studies with multiple arms evaluating the same treatment type, we pooled the mean and standard deviation data from these arms, effectively treating them as a single treatment group [13]. Trials that used intention-to-treat analysis for post-intervention measurement of non-adherent trials were extracted first, otherwise patients who adhered to the intervention were included. When relevant data for imputation was not available in the article, an email request for measurement results details was sent to the corresponding author. If the corresponding author did not respond, the trial was only included for review and not included in the meta-analysis. Information extraction was performed by two independent researchers in a previously created electronic Excel form, discrepancies between researchers were reviewed and resolved by a third reviewer. The agreement between two reviewers was evaluated by the Cohen’s Kappa coefficient, of which κ > 0.8 (almost perfect), κ > 0.6 (substantial), κ > 0.4 (moderate), κ > 0.2 (fair), 0 < κ < 0.2 (slight poor), κ < 0 (poor) [14].

### Risk of bias and CINeMA assessment

Cochrane Risk of Bias 2.0 (RoB2) tool was applied for the assessment of all trials [15]. There are five domains in this tool: randomization process, deviations from planned interventions (effect of adhering to intervention and assignment to intervention), missing outcome data, outcome measurement, and selection of reported outcome. The outcome depended on the assessment of each area in three categories (i.e., “low risk of bias”, “some concerns”, “high risk of bias”). An algorithm built into the tool can generate the overall risk of bias based on the three categories according to the following rule: (1) low risk of bias – all areas were classified as low risk; (2) some concerns - at least one domain was identified as problematic, but the remaining domains were not identified as high risk; (3) high risk of bias - at least one area was identified as high risk or concerns were identified in all areas. A review author made the final decision in cases where there was disagreement after two independent researchers completed the risk of bias assessment.

The CINeMA assessment, conducted using online software, evaluated the credibility of evidence for both direct and indirect comparisons between non-pharmacological interventions and comparator [16]. This assessment tool incorporates six domains: within-study bias, reporting bias, indirectness, imprecision, heterogeneity, and incoherence. (1) Within-study bias refers to the risk of bias within individual studies, as previously discussed; (2) Reporting bias considers the potential for both suspected and undetected biases; (3) Indirectness assesses the relevance of study evidence to the research question; (4) Imprecision evaluates the impact of minimal clinically important differences (MCID) on the estimated treatment effects. Following the recommendations of previous publications, we defined the MCID for pain intensity as 2 points, for functional disability as 16 points, and overall efficacy as 0.7 [17,18]; (5) Heterogeneity examines the influence of variability across studies on the confidence intervals of treatment effect estimates; (6) Incoherence compares the consistency between direct and indirect evidence regarding treatment effects. The assessment results for each domain were categorized into three levels: ‘no concerns’, ‘some concerns’, and ‘major concerns’.

### Data analysis

In this research, we utilized the ‘BUGSnet’ package for NMA, which is a package from the R statistics software version 4.3.2 (The R Foundation, Vienna, Austria). Because of the clinical heterogeneity in the selected trials, a random effects model was employed. This assumed that each study was estimating distinct yet related intervention effects. Given the size and complexity of the networks analyzed in this study, network diagrams were presented in a tabular format for clarity and conciseness. Interventions included in the NMA must form a connected network. Interventions with no connections to the primary network, and therefore no indirect comparisons to the chosen comparator, were excluded. The random effects model employed Bayesian NMA. We utilized a Markov Chain Monte Carlo (MCMC) algorithm with three chains, each running for 25000 iterations. A burn-in period of 5000 iterations was discarded. Convergence of the MCMC chains was assessed using the Gelman-Rubin diagnostic test, with a threshold of 1.05 for acceptance, as recommended by van Valkenhoef et al. [19]. Convergence was further assessed using Geweke diagnostic tests. These tests compare the mean of early and late parts within each Markov chain. Z-scores outside the range of -1.96 to 1.96 suggest potential convergence issues [20]. When convergence problems were detected, the model was re-run with a larger number of iterations to improve specification [21]. Intervention effects regarding to the pain intensity and functional disability were determined by comparing the post-pre change in scores between intervention groups. We calculated the mean and standard deviation (SD) of the change in scores for each intervention group. The SD of the change in scores for each group was calculated using the pre- and post-test SDs and the formula provided by Deeks et al. [22]. For pain intensity measurements, a negative relative effect (mean difference of post-pre change) indicates that the experimental group is more effective than the comparator. Conversely, for functional disability measurements, a positive relative effect suggests that the experimental group is more effective. The overall efficacy was determined by calculating the relative risk (RR), which is the ratio of ineffective cases in the experimental group divided by the ratio of ineffective cases in the comparator. A RR less than one signifies that there are fewer ineffective cases in the experimental group compared with the control group. The effectiveness of each intervention was estimated using 95% confidence intervals (CIs) for the relative effect compared to a common comparator, ‘medications’. Results were presented by forest plot, surface under the cumulative ranking curve (SUCRA) table, and the pairwise comparison table (or league table). The forest plot and pairwise comparison table displayed the relative effectiveness of each intervention, while a sucra table ranked the interventions based on their probability of being the most effective. Heterogeneity was considered using the Cochran Q test (p < 0.05), supplemented by the I^2^ statistic with 95% CIs. A low level of heterogeneity was represented by 25% < I^2^ < 50%, while a moderate level was represented by 50% < I^2^ < 75%, and a substantial level was represented by an I^2^-value greater than 75% [23]. To investigate potential sources of heterogeneity, we conducted a meta-regression analysis. This analysis examined the influence of several potential moderators, including LBP subtype, age, intervention duration, sample size, and overall bias. LBP subtype was noted as the symptom for diagnosis, which included non-specific LBP and specific LBP. Age-related demographic data was categorized into two groups: adults, which encompasses individuals aged between 18 and 65, and the elderly, signifying those who are older than 65. Intervention duration and sample size were entered as the number of weeks and number of participants, respectively. Overall bias was categorized into ‘low’, ‘medium’, and ‘high’. Sensitivity analyses were conducted to compare the model fit of fixed and random effects models. We assessed model fit by examining the leverage of each data point against its contribution to the total posterior deviance in the model fit plot [21]. Data points lying outside the second parabola from the edge of the plot were considered as poor model fit. Data points with high leverage values were considered influential. A well-fitting model should exhibit a relatively uniform distribution of influential points. Furthermore, model comparison was based on the Deviance Information Criterion (DIC). A lower DIC generally indicates a better fit. Additionally, the global measure ($\overset{―}{D}$res) was evaluated, with values closer to the number of intervention arms in the model suggesting a better fit [24]. The consistency assumption was evaluated by comparing an inconsistency model with a consistency model, both of which were assessed using the model fit plot. This comparison aimed to assess the agreement between direct and indirect evidence within the network of interventions [25]. The study’s overall quality was evaluated based on its risk of bias assessment. Publication bias was evaluated using a contoured enhanced funnel plot and Egger’s test. The asymmetry of studies in a funnel plot, revealing a significant difference in p-value below 0.05, suggests the existence of publication bias.

## Results

**Fig 1:**
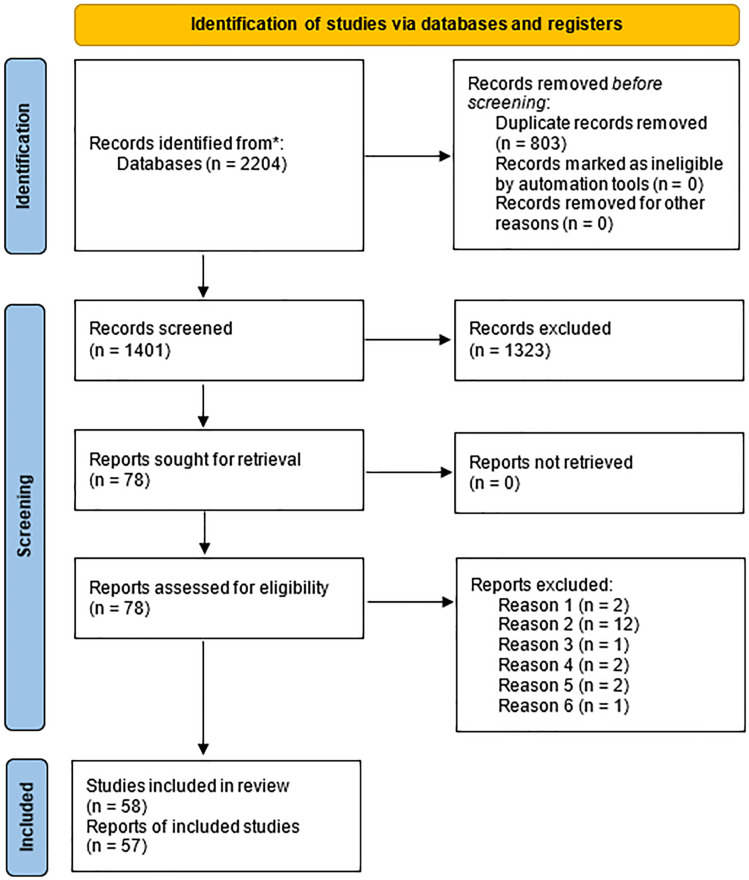
Flowchart for the identification of new studies through databases and registers according to PRISMA. Reason 1: no response from corresponding author; Reason 2: retro-spective study; Reason 3: involving intravenous therapy; Reason 4; involving lumbar spinal surgery; Reason 5: not carry out in China; Reason 6: use healthy control without report of risk control.

After searching databases and removing duplicates, 1401 articles were identified. Seventy-eight articles met the inclusion criteria and 57 trials (5558 patients) were reviewed for analysis after exclusion (Fig 1). Agreement between reviewers was excellent (κ = 0.93).

### Study chracteristics

According to the outcome measurement of the included trials, 55 trials reported pain intensity [26–81], 48 trials reported functional disability [26–46,48–51,54,55,57–64,67,71–77,79–83], 16 trials reported overall efficacy [26,27,31,36,41–43,44,50,51,53,57,70,71,79,82], 6 trials reported quality of life [26,38,41,42,60,64], 7 trials reported mental health [33,34,37,40,47,49,64], 19 trials reported physical performance [28,29,38,39,41,42,47,48,52,54,56,60,63,64,67,68,72,76,80], and 19 trials reported general health [33,35,37,40,42,44,46–51,53,55,63,66,72,78,81]. The most used instruments to measure pain intensity were the visual analogue scale and the numeric rating scale. For functional disability measurement, Oswestry disability index, Rolland-Morris disability questionnaire, and Japanese Orthopedic Association Scale were commonly seen. Improvement in symptoms was often used to assess the overall efficacy of an intervention. We also found tools in measurement for quality of life (e.g., GQOL-74, OA-QAOL, WHOQOL-BREF), mental health (e.g., PHQ-9, GAD-7, PCS, PSEQ), physical performance (e.g., ASLR, trunk ROM, IMS), general health (e.g., SF-36, HR, BP, sleep quality, inflammatory cytokines) (S3 Table). The selection of outcomes for NMA was guided by the need for consistent measurement. Therefore, only pain intensity, functional disability, and overall efficacy were included, as their respective measurement tools demonstrated the highest degree of similarity.

Although there were differences in the type of pain and symptoms between trials, the included trials can generally be divided into LBP with specific LBP (k = 29) and non-specific LBP (k = 28). Heterogeneity can be observed in the LBP and NSLBP subtypes, and the symptoms of these pains were also different (S3 Table). One trial exclusively included male patients [27], and five trials neglected to disclose the sex ratio [30,56,60,61,69]. There was one trial that included only elderly patients [74,75].

Non-pharmacological approaches from China mostly developed from traditional Chinese medicine or traditional Chinese exercise. We found that some intervention groups involved the use of only one type of therapy, such as acupuncture [26,32,36,44,46,51,55,59,65,79,80,82], Chinese massage (Tuina or acupoint massage) [31,35,39,46,56,58,62], auricular or hand acupoint acupuncture or acupressure [51,55,74,75], cupping and scraping [27], moxibustion [50,59,82,83], TENS [60,70], PENS [43,71], traction [50,73], traditional Chinese exercise (Baduanjin exercise, Wuqinxi exercise, or Taichi exercise) [29,47,52,73], lumbar dynamic exercise [33,37,47,63], core stability exercise [28,49,52,54,60,71,81], extracorporeal shock wave therapy [34], light therapy [64], and surgery [78]. The remaining approaches originated from combined therapies. Most involved combination of the single therapy [26,30,31,39,40,44,50,53,54,57,58,60–62,71,72,81], while others combined interventions such as usual care [38,53,66,76], medications [40,41,48,77,83], mental related treatment [37,49,63,72], thermos [32,33,41,45], and ointment [68]. Some combinations have more than two therapies [40–42,45,53,67,76].

Assessment time points followed the general “pre-intervention” and “post-intervention” format. Some trials conducted assessment during the intervention (18 trials) [27,30,35,36,38,40,45,47,49,54,58,61,62,65,66,68,74,75], and had follow-up assessment post-intervention (26 trials) [28,31–34,37,40,42,46,48,49,51,54,57,61,62,65,68,72–78,80,83]. Four trials were conducted for the post-intervention assessment some time after the end of the intervention instead of immediately [32,51,76,78]. One trial involved a pre- and post-treatment daily [69].

### Risk of bias and CINeMA assessment

**Fig 2:**
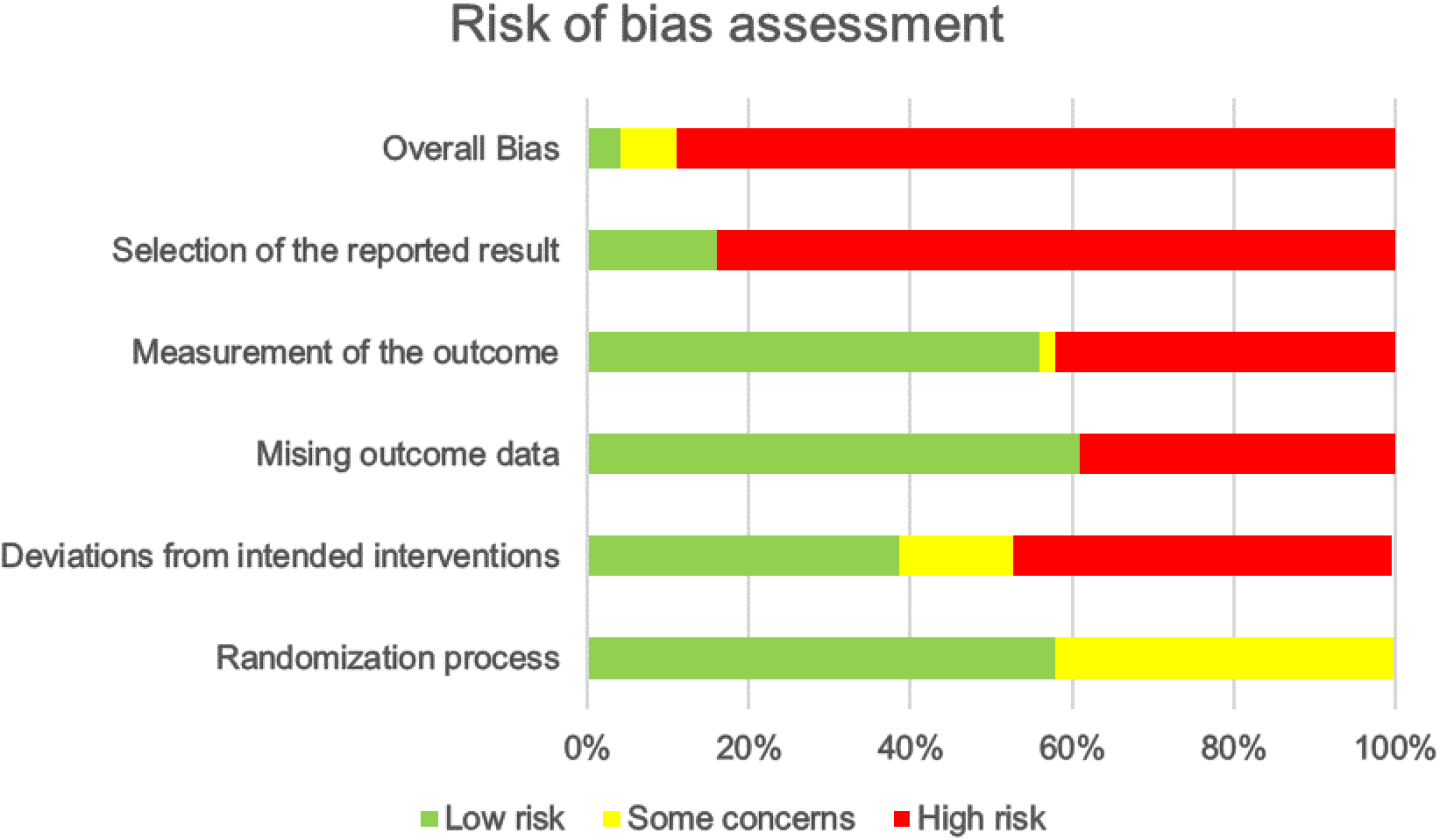
Risk of bias assessment.

Our risk of bias assessment revealed that 89% of the studies exhibited high risk (Fig 2). The most concerning issue was the selection of reported results (84%), as these studies relied solely on subjective questionnaires for outcome measurement. No additional assessments were conducted during or after the intervention, and the rationale for the intervention duration remained unclear.

The second major concern was deviations from intended interventions (47%). Many studies lacked clarity regarding personnel involved in randomization and group assignment, raising concerns about potential biases. For example, caregivers and intervention providers may have known the assigned groups beforehand. Furthermore, only 9 trials employed intention-to-treat analysis (S4 Table), potentially impacting the outcomes due to non-adherence by some participants.

The third significant risk factor was outcome measurement. In some cases, the assessors may also involve in delivering the intervention, potentially introducing bias. Additionally, when the intervention methods did not differ significantly from the comparators, relying solely on subjective questionnaires for outcome assessment could be influenced by participants’ knowledge of the received intervention.

Finally, missing outcome data posed a substantial high risk. Since many studies used per-protocol analysis, patients lost to follow-up could significantly impact the results, and researchers did not adequately address this potential bias.

Due to the high volume of indirect evidence for pairwise comparisons between non-pharmacological interventions and medications, only the domains of imprecision and heterogeneity were considered to evaluate the confidence of the meta-analysis results. Evidence assessed as having ‘no concern’ or ‘some concern’ for these domains was included in the subsequent analysis. Results were listed in the S5 Table.

### Pain intensity

**Fig 3:**
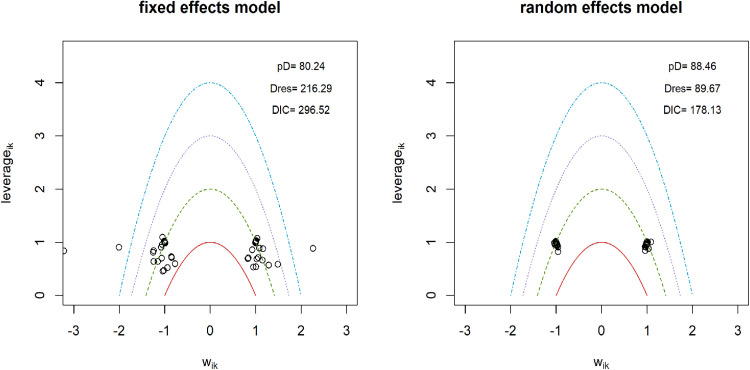
Sensitivity analysis for models data fit in pain intensity.

**Fig 4:**
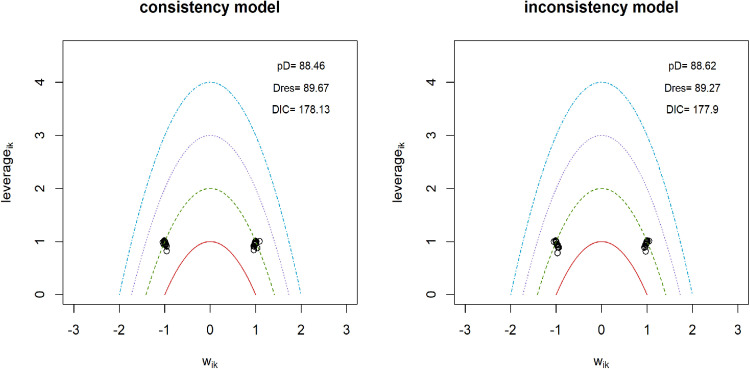
Inconsistency analysis for models data fit in pain intensity.

**Fig 5:**
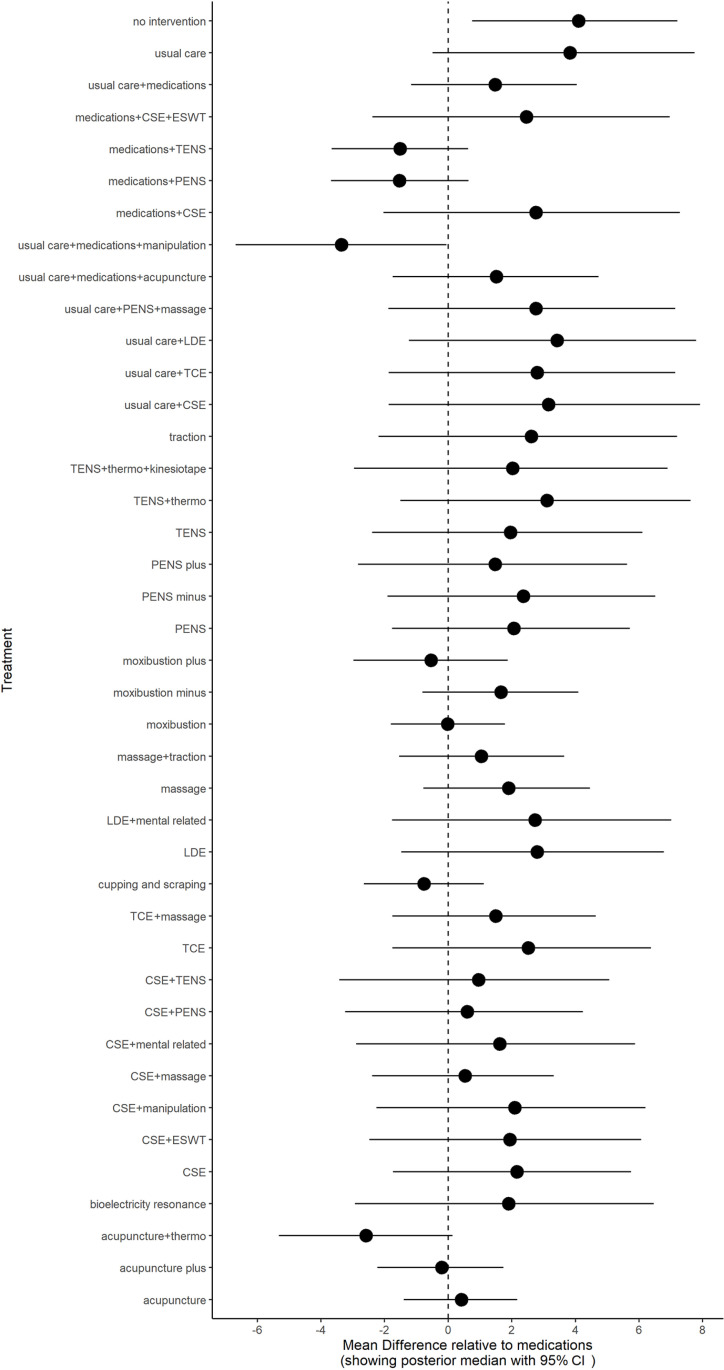
Forest plot for pain intensity.

Network diagrams illustrated the presence of 42 distinct interventions within the network used to analyze pain intensity (Table 1). Direct evidence within this network supported only 7% of all possible pairwise comparisons between these interventions. In the random effects model of the Bayesian NMA, results from the Gelman-Rubin and Geweke diagnostic tests indicated that the MCMC chains had converged (S6 Table). It displayed a substantial level of heterogeneity: Q-value = 136.04, I^2^ = 92.6% (95% CIs: 88.8% - 95.2%). Meta-regression analysis, examining the influence of LBP subtype, intervention duration, sample size, and overall bias failed to identify a significant source of heterogeneity. Age was excluded as a moderator in the meta-regression analysis to avoid the potential for aggregation bias, given that most included studies recruited adults within the same age range. Sensitivity analysis indicated that the random effects model provided more robust and reliable estimates compared to the fixed effects model (Fig 3). The model fit plot for the inconsistency analysis indicated that both the consistency and inconsistency models exhibited similar fit (Fig 4). This finding suggests that the assumption of consistency within the NMA is not violated. The forest plot revealed that most non-pharmacological treatments did not demonstrate superior pain reduction (relative effect median and range < 0) compared with medications alone at the end of treatment (Fig 5). When non-pharmacological interventions were combined with medications, the relative effectiveness was uncertain. Notably, TENS, PENS, usual care and manipulation in conjunction with medications appeared to significantly reduce pain intensity. Conversely, CSE, CSE combined with ESWT, and acupuncture in conjunction with medications did not show any significant improvement in pain reduction. Based on probability estimates, SUCRA table ranked ‘no intervention’ and ‘usual care’ treatments as least effective (S7 excel table). The table ranked the following five treatments as most effective: ‘usual care + medications + manipulation’, ‘acupuncture + thermo’, ‘medications + PENS’, ‘medications + TENS’, and ‘cupping and scraping’. ‘medications’ were ranked higher than most of treatment in the table. Analysis in league table revealed that medications were significantly more effective than ‘no intervention’ in reducing pain (S8 excel table). Adding usual care and manipulation to medications improved pain outcomes. ‘usual care + medication + manipulation’, as well as ‘acupuncture + thermo’, were among the most effective treatments for pain reduction. Results also showed that only ‘no intervention’ had no significant impact on pain levels compared with most other treatments. All positive reports exhibited moderate to high confidence regarding imprecision, and high heterogeneity had minimal impact on these findings (S5 Table). Only five studies directly compared medications to other treatments. Evidence of publication bias was apparent, likely due to the limited number of studies. Consequently, a funnel plot analysis was not applicable in this case.

**Table 1:** Network digrams for pain intensity, functional disability, and overall efficacy.

Network diagram characteristic	value		
Pain intensity	Functional disability	Overall efficacy
Number of Interventions	42	18	9
Number of Studies	39	18	8
Total Number of Patients in Network	3793	2463	1065
Total Possible Pairwise Comparisons	861	153	36
Total Number of Pairwise Comparisons With Direct Data	56	24	10
Is the network connected?	TRUE	TRUE	TRUE
Number of Two-arm Studies	29	13	6
Number of Multi-Arms Studies	10	5	2

### Functional disability

**Fig 6:**
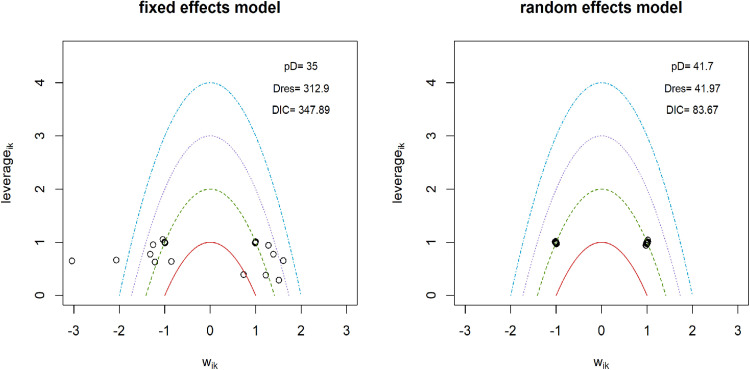
Sensitivity analysis for models data fit in functional disability.

**Fig 7:**
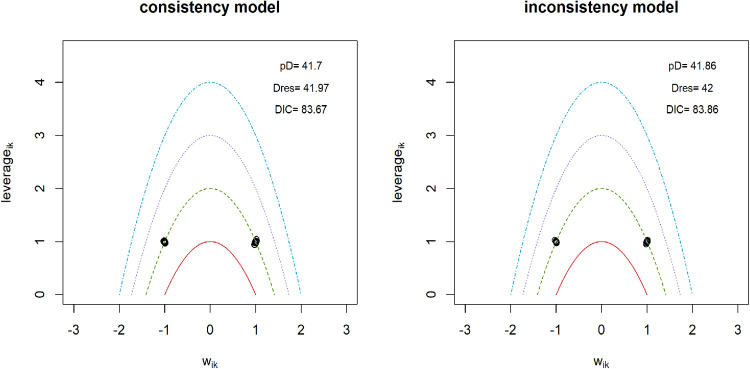
Inconsistency analysis for models data fit in functional disability.

**Fig 8:**
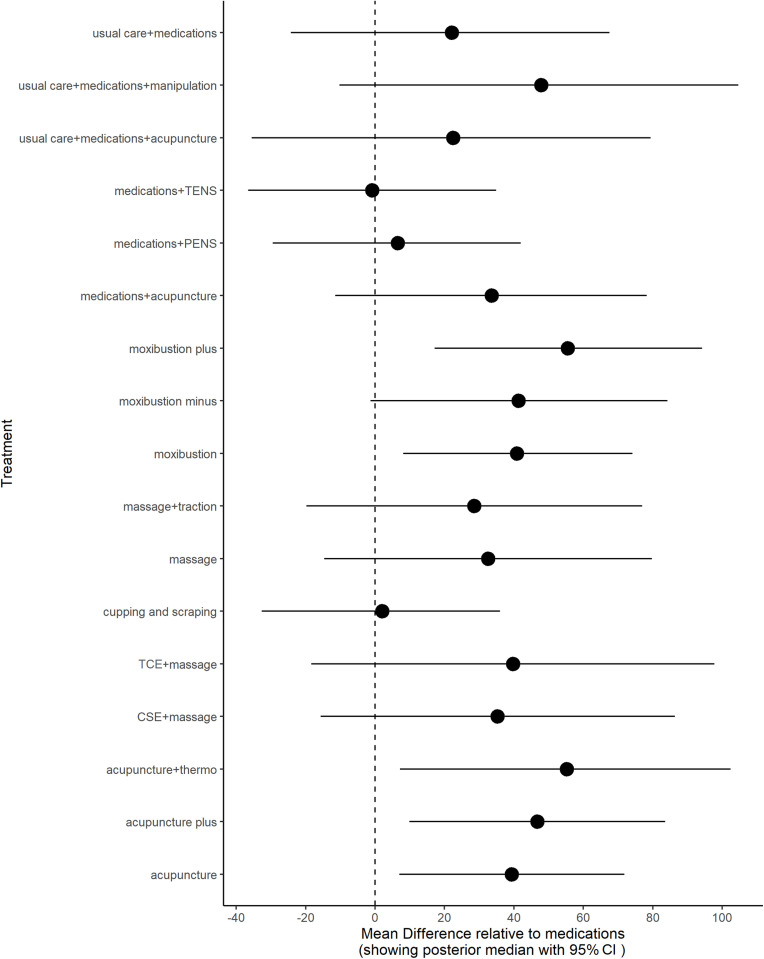
Forest plot for functional disability.

To construct a connected network for comparing non-pharmacological interventions with pharmaceuticals in the analysis of functional disability, only 18 interventions were included (Table 1). Direct evidence was available for 16% of all possible pairwise comparisons. A random effects model was employed, and its reliability was confirmed by successful convergence, as indicated by the Gelman-Rubin and Geweke diagnostic tests (S9 table). Significant heterogeneity was detected, as evidenced by a substantial level of Q-value of 277.82, I^2^ = 97.5% (95% CIs: 96.4% - 98.2%). Meta-regression analysis revealed that only the subtype of LBP could explain 12.51% of the observed heterogeneity, the rest of moderators failed to identify the source of heterogeneity. Sensitivity analyses favored the random effects model, suggesting it produced more robust and reliable estimates than the fixed effects model (Fig 6). The model fit plot from the inconsistency analysis showed comparable fit for both consistency and inconsistency models (Fig 7). This finding supports the assumption of consistency within the NMA. Therapies that included acupuncture, massage, and moxibustion demonstrated superior treatment effects compared to medications alone (Fig 8). When medications were combined with other therapies, TENS and PENS did not show superior treatment effects compared to combinations involving acupuncture, manipulation, or usual care. The SUCRA rank table placed ‘medications + TENS’, ‘medication + PENS’, ‘medications’, and ‘usual care + medications’ among the bottom five ranked therapies. This indicates that these treatment options demonstrated the least effectiveness in improving functional disability compared to other therapies in the network (S10 excel table). The imprecision assessment identified ‘major concerns’ for ‘medications + PENS’ and ‘medications + TENS’ (S5 Table). The league table reveals that acupuncture and moxibustion related therapies, when used as single therapies or combined therapies, demonstrated significantly better treatment outcomes for functional disability compared to medications alone (S11 excel table). When medications were combined with other therapies, including manipulation, TENS, PENS, acupuncture, usual care, the effectiveness of these combinations was uncertain. Among these therapies, ‘acupuncture + usual care + medications,’ for which the imprecision assessment raised ‘major concerns’. The treatment effects of ‘CSE + massage’ and ‘TCE + massage’ were also uncertain when compared to medications. High heterogeneity had minimal influence on the confidence interval for functional disability. Similar to the limited number of pairwise comparisons between medications and other therapies observed for pain intensity analysis, funnel plots were also not available for the analysis of functional disability.

### Overall efficacy

**Fig 9:**
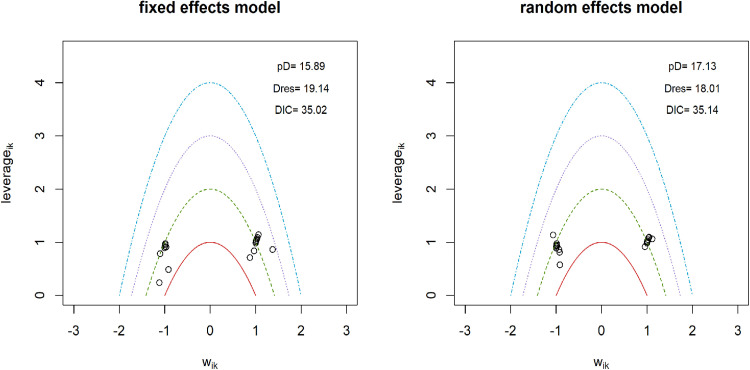
Sensitivity analysis for models data fit in overall efficacy.

**Fig 10:**
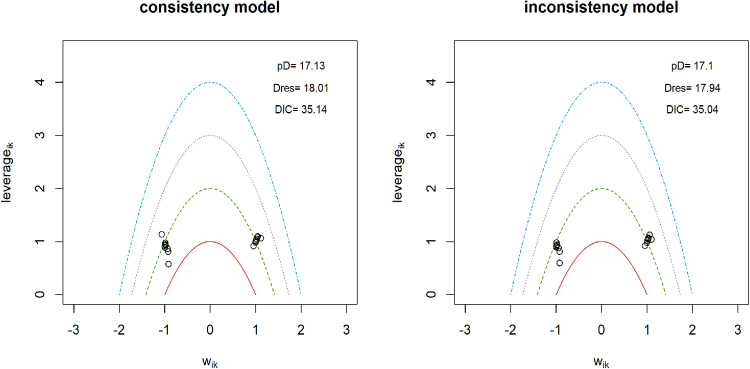
Inconsistency analysis for models data fit in overall efficacy.

**Fig 11:**
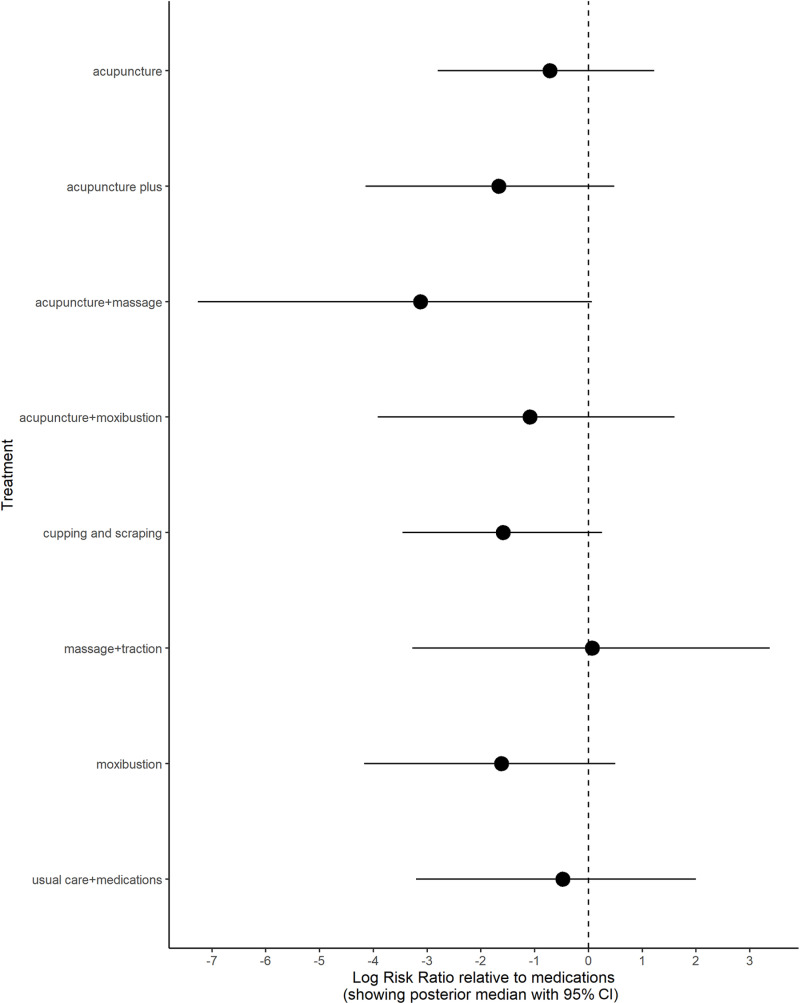
Forest plot for overall efficacy.

Nine interventions were identified for the overall efficacy analysis to establish a connected network for comparing medications with other therapies (Table 1). Direct evidence was available for approximately 28% of all possible pairwise comparisons within this network. The random effects model was implemented, and its performance was validated through successful convergence. This was confirmed by the Gelman-Rubin and Geweke diagnostic tests (S12 table). The wide 95% CIs (Q-value = 1.72, I^2^ = 0% (95% CIs: 0% - 89.6%) for heterogeneity suggested significant uncertainty regarding the true extent of heterogeneity among studies. Furthermore, meta-regression analysis was unable to identify any potential sources that could explain the observed heterogeneity. Sensitivity analyses demonstrated that the random effects model yielded more robust and reliable estimates than the fixed effects model (Fig 9). The model fit plot (Fig 10) from the inconsistency analysis demonstrated comparable fit for both consistency and inconsistency models, providing evidence for the assumption of consistency within the NMA. The forest plot revealed that individual treatments like acupuncture alone, cupping or scraping, or moxibustion, generally lead to fewer treatment failures than medications. Combining acupuncture with massage or moxibustion appeared to be more effective overall than medication. The effectiveness of massage combined with traction was unclear. When medications were used alongside usual care, they seemed to result in fewer treatment failures compared to medications alone (Fig 11). The SUCRA rank table indicated that medication alone was ranked 8th in effectiveness (S13 excel table). This is more favorable than ‘massage + traction’, which likely ranked lower. However, treatments like acupuncture-related therapies, ‘cupping + scraping’, moxibustion, and ‘usual care + medications’ achieved higher rankings than medication used independently. The league table revealed that only ‘acupuncture + massage’, ‘acupuncture plus’, ‘moxibustion’, and ‘cupping and scraping’ demonstrated a lower rate of ineffective cases close to significant level compared to medication alone (S14 excel table). Imprecision assessments indicated moderate to high confidence for treatment of ‘cupping and scraping’, ‘moxibustion’, ‘acupuncture plus’, and ‘acupuncture + massage’. Notably, only the treatment effect of ‘moxibustion’ remained unaffected by high levels of heterogeneity. A funnel plot could not be generated owing to insufficient direct comparisons between medication and alternative therapies. The raw data extracted were compiled in **S15 Excel table**. **S16 Excel table** contained the exclusion reasons for all identified studies. The risk of bias assessment results were displayed in **S17 Excel table**, while **S19 Excel table** outlined the exclusion criteria for all included studies.

## Discussion

This review employed Bayesian NMA to compare the effectiveness of non-pharmacological interventions and medications for LBP in Chinese adults. Three separate networks were constructed for pain intensity, functional disability, and overall efficacy, due to varying reporting rates. A random-effects model, incorporating sensitivity and transitivity assumptions, was fitted for each outcome. Substantial heterogeneity was observed across studies. Meta-regression revealed that LBP subtype partially explained heterogeneity in functional disability, but the sources of heterogeneity for pain intensity and overall efficacy remained unidentified. Limited study numbers for each treatment, diverse treatment methods, and high ratio of overall bias likely contributed to the heterogeneity.

Based on forest plots, SUCRA rankings, and pairwise comparisons, medications demonstrated superior effectiveness for pain intensity compared to most non-pharmacological interventions. Acupuncture and moxibustion within TCM exhibited better outcomes for functional disability and overall efficacy than medications alone. Caution is advised regarding “medications + PENS” and “medications + TENS” combinations. While potentially effective for pain relief, these combinations may be less beneficial for improving functional disability. In most cases, combinations of TCM therapies demonstrated superior outcomes for pain intensity, functional disability, and overall efficacy compared to combinations of medications with other therapies. The effectiveness for functional disability may be affected by subtypes of LBP. In addition, the evidence for pain intensity and functional disability was considered more credible than the evidence for overall efficacy.

Non-pharmacological intervention was preferred to treat LBP based on previous published reviews [84–92]. Both non-specific and specific LBP were included in some of the reviews [85,88,92]. The interventions for non-specific LBP were the focus of the remaining studies. Non-pharmacological intervention was found to be effective for both types of LBP in improving pain intensity, disability, and overall efficacy, but our review disagreed with these earlier findings. Medications alone were more effective for pain relief than most of non-pharmacological interventions, while non-pharmacological interventions demonstrated superior outcomes for functional disability and overall efficacy. This difference arises from the limited direct comparisons between non-pharmacological interventions and medications in the included studies [87,92]. Studies investigating non-pharmacological interventions often employed control groups that included a mix of pharmaceuticals, usual care, placebo, and sham treatments. This heterogeneity in control groups precluded the use of conventional non-NMA for direct comparisons between non-pharmacological interventions and medications. Our findings are in agreement with the report by Cashin et al. [93], which primarily examined pharmaceutical interventions and found them to be more effective in reducing pain intensity than in improving functional disability or enhancing overall efficacy. Despite this, acupuncture combined with thermal therapy exhibited superior efficacy compared to medications alone. Furthermore, combining medications with TENS, PENS, or ‘usual care + manipulation’ also resulted in improved treatment outcomes. The multifaceted nature of LBP necessitates a multicomponent management approach [94]. Our findings regarding pain intensity treatment support this concept. However, within the realm of combined non-pharmacological interventions, psychological and exercise-based therapies did not demonstrate superior treatment effects, suggesting that the characteristics of the pain condition may significantly influence treatment outcomes.

While the confidence of results was primarily assessed based on imprecision and heterogeneity from the CINeMA framework, the superior treatment effects for functional disability and overall efficacy observed for non-pharmacological interventions were largely restricted to moxibustion. In addition, acupuncture-related therapies were anticipated to demonstrate better treatment effects on disability only compared to medications. Previous findings with direct evidence support our observation regarding the efficacy of acupuncture [95]. However, the superior efficacy of these effects observed in our study should be interpreted cautiously due to the high risk of overall bias and the incomplete incoherence assessment.

This study observed a predominance of Traditional Chinese Medicine (TCM) interventions within the non-pharmacological treatment. This prevalence is likely influenced by sociocultural factors, including patient beliefs and the prominent role of TCM within the healthcare system. Studies have shown that a deeper understanding of TCM principles is associated with improved treatment outcomes [96]. This cultural context fosters a strong belief in the efficacy of TCM for LBP among patients, leading to repeated treatment and intergenerational transmission of these practices. From a national perspective, the large market demand for TCM, coupled with an aging population that exhibits a strong preference for TCM over Western medications, has stimulated significant research and regulatory support for its use [97]. Notably, existing regulations often emphasize the potential of TCM to improve overall health rather than solely focusing on disease treatment. The high risk of overall bias, particularly evident in non-pharmacological RCTs from this study with inadequate reporting of objective outcome measures, aligns with current scientific comment on TCM for LBP.

Despite the inherent flexibility of Bayesian NMA in accommodating heterogeneity, the high risk of overall bias in existing studies necessitates careful consideration for future research on non-pharmacological interventions for LBP in China. To enhance methodological rigor, we recommend the following: (1) When dealing with significant missing data, prioritize “intention-to-treat” analysis over “per-protocol” analysis; (2) Implement proper blinding procedures to mitigate biases such as concealment and selective reporting [98]; (3) Regarding to the selection of control group, consider using time-to-attention controls, sham controls, placebo controls, or active controls with well-defined adverse events and risk ratios. For interventions combining non-pharmacological methods with existing therapies, use the existing therapy as the comparator; (4) Conduct multiple assessments throughout the intervention period to determine the minimal intervention duration required for symptom improvement. Additionally, include post-intervention assessments to evaluate the sustained effects of the intervention. (5) Beyond pain intensity, functional disability, and overall efficacy, include assessments of psychological health and physical performance, particularly in chronic LBP studies due to the multidimensional nature of the condition [99]; (6) Incorporate objective assessment measures alongside subjective assessments to minimize the risk of investigator bias. Weak blinding techniques can potentially influence the questionnaire process.

While our findings generally support the use of non-pharmacological interventions for improving functional disability and overall efficacy, current research is the lack of cost-effectiveness assessments for these interventions. Buchbinder et al. published an article advocating for the elimination of low-value treatment for LBP [100]. High-income nations bear a significant financial burden when it comes to LBP intervention [101]. Despite a downward trend in the cost per patient for low- and middle-income nations [102], our experience shows that spending more than $1,000 is still shocking. Due to the pressure of an aging population and limited healthcare resources [103], it became imperative to assess the cost-effectiveness of non-pharmacological interventions for LBP.

The limitations of our study should be noted. We simply separated LBP into types that are specific and non-specific. As a result, the findings cannot be used to explain how a particular non-pharmacological intervention affects the improvement of a particular kind of LBP. Even though we discovered that many studies used follow-up after the intervention ended, we only retracted data from the post-intervention. As such, it was unable to disclose the long-term impact of the non-pharmacological intervention. Moreover, retracted data on dosage, exercise volume, and intensity could not be investigated for generalization because of variations in implementation and the limited reporting of each intervention. Most importantly, the validity of this study may be limited by the complex network filled with high volume of indirect evidence and a limited number of reports for each intervention, despite the robustness of the Bayesian NMA.

## Conclusions

This study aimed to systematically review and conduct a Bayesian NMA of RCTs in Chinese adults with LBP to determine the relative effectiveness of non-pharmacological interventions compared to medications. Our findings suggest that: (1) pharmaceuticals alone, or in combination with non-pharmacological interventions, generally demonstrated superior efficacy for pain intensity compared to most non-pharmacological interventions; (2) non-pharmacological interventions may offer greater benefits for disability and overall efficacy. Substantial heterogeneity was observed across studies. While its impact on pain intensity and functional disability assessments was limited, it significantly influenced the confidence in overall efficacy estimates with major concerns of imprecision. Heterogeneity may be attributed to LBP subtypes in the case of functional disability, but the sources of heterogeneity for pain intensity and overall efficacy remain unidentified. Given the high percentage of studies with a high risk of overall bias, the results should be interpreted with caution.

## Supporting information

S1 Table Search strategy of database. (DOCX)

S2 Protocol Study procotol. (PDF)

S3 Table Characteristics of included studies. Adr, adrenaline; ASLR, angle of straight leg raising; β-EP, beta-endorphins; bid, twice a day; BMI, body mass index; BP, blood pressure; BPI-SF, brief pain inventory-short form; BSS, biodex stability system; CLBP, chronic low back pain; CNSLBP, chronic non-specific low back pain; CSE, core stability exercise; C-SF-MPQ, Chinese short-form of McGill pain questionnaire; C-SFODI, simplified Chinese version of the Oswestry Disability Index; CTR, control group; d, day; DAL, daily activity level; dep, dependent; DLBP, discogenic low back pain; DLSS, degenerative lumbar spinal stenosis; DMS, deep muscle stimulation; EA, electroacupuncture; EAS, electronic acupuncture shoes; exerc, exercise; ESWT, extracorporeal shock wave therapy; EXP, experimental group; F, female; FABQ, fear avoidance beliefs questionnaire; FAI, Frenchay activity index; 5-HT, 5-hydroxytryptamine; GAD-7, 7-item generalized anxiety disorder scale; GQOL-74, generic quality of life inventory-74; HADS, hospital anxiety and depression scale; H-MRS, hydrogen-1 magnetic resonance spectroscopy; HR, heart rate; HRV, heart rate variability; IL, interleukin; IMS, isometric muscle strength; int, intervention; JOA, Japanese Orthopaedic Association score; LBP, low back pain; LDD, lumbosacral disc degeneration; LDE, lumbar dynamic exercise; LDH, lumbar disc herniation; LPL, low power laser; M, male; m, mean; mALBPDS, modified Aberdeen low back pain disability scale; MCID, minimal clinical important difference; MCS, mental component summary; MET, motivational enhancement therapy; MFI, multidimensional fatigue inventory; min, minute; mJOA, modified Japanese Orthopaedic Association score; mo, month; mODI, modified Oswestry disability index; NA, not applicable; NE, norepinephrine; NRS, numeric rating scale; NSAIDs, nonsteroidal anti-inflammatory drugs; NSLBP, non-specific low back pain; OA-QALQ, osteoarthritis quality of life questionnaire; ODI, Oswestry disability index; PASS, pain anxiety symptom scale; PCS^*^, pain catastrophizing scale; PCS^**^, physical component summary; PENS, percutaneous electrical nerve stimulation; PHQ-9, 9-item patient health questionnaire; PPI, present pain intensity; PRI, pain rating index; PSEQ, pain self-efficacy questionnaire; PSQI, Pittsburgh sleep quality index; pts, points; q2d, every 2 day; q3d, every 3 day; q4d, every 4 day; QBPDS, Quebec back pain disability scale; qd, once a day; qod, every other day; RMDQ, Roland-Morris disability questionnaire; ROM, range of motion; ROS, reactive oxygen species; SANSLBP, sub-acute non-specific low back pain; SAS, self-rating anxiety scale; SCS, self-compassion scale; SCT, self-compassion therapy; SDS, Zung self-rating depression scale; sEMG, surface electromyography; sess, session; SF-12, quality of life 12-item short form survey; SF-36, 36-item short form health survey; SF-MPQ, short-form of McGill pain questionnaire; std, standard deviation; TCE, traditional Chinese exercise; TCMS, traditional Chinese medicine syndrome integral scale; TENS, transcutaneous electrical nerve stimulation; tid, three times a day; TNF-α, tumor necrosis factor-α; TSK, Tampa scale of kinesiophobia; VAS, visual analog scale; w, week; WHOQOL-BREF, the world health organization quality of life scale; yr, year.
(DOCX)

S4 Table Risk of bias assessment. (DOCX)

S5 Table Imprecision and heterogeneity assessment from CINeMA. (DOCX)

S6 Table Covergence results of random effects model for pain intensity. (DOCX)

S7 excel table SUCRA rank of treatment effect for pain intensity. (XLSX)

S8 excel table League table of pairwise comparison between treatment for pain intensity. (XLSX)

S9 Table Covergence results of random effects model for functional disability. (DOCX)

S10 excel table SUCRA rank of treatment effect for functional disability. (XLSX)

S11 excel table League table of pairwise comparison between treatment for functional disability. (XLSX)

S12 Table Covergence results of random effects model for overall efficacy. (DOCX)

S13 excel table SUCRA rank of treatment effect for overall efficacy. (XLSX)

S14 excel table League table of pairwise comparison between treatment for overall efficacy. (XLSX)

S15 excel table Bayesian NMA dataset. (XLSX)

S16 excel table Eligiblility and exclusion criteria for identified studies. (XLSX)

S17 excel table Risk of bias assessment. (XLSX)

S18 PRISMA checklist. (PDF)

S19 excel table Exclusion criteria for all included studies. (XLSX)
