# Supplementary material for: The effectiveness of non-pharmacological interventions for low back pain in China: A systematic review and network meta-analysis
Source: PLoS One. 2025 May 9;20(5):e0322929. doi: 10.1371/journal.pone.0322929 (PMC12063812; doi:10.1371/journal.pone.0322929)
Supplement: S1 Table — (DOCX) [file pone.0322929.s001.docx]

| **Database** | **Keywords and connected method** |
| --- | --- |
| The Cochrane Library | #1 [back pain] **explode all trees**  #2 (“low back pain” or “low back ache” or “low backache” or “low back injury” or “low back disorder” or “lumbago” or “sciatica” or “spondylosis”):**ti,ab,kw in Trials -(Word variations have been searched)**  #3 (“nonpharmacological” or “advice” or “education” or “thermos therapy” or “heat” or “hot” or “cold” or “bath” or “sauna” or “fumigation” or “steaming” or “spa” or “manual therapy” or “massage” or “mobilization” or “manipulation” or “exercise” or “Qi Gong” or “Tai Chi” or “postural therapy” or “traction” or “tape” or “kinesiotape” or “patch” or “band” or “electrotherapy” or “laser therapy” or “TENS” or “PENS” or “shortwave diathermy” or “ultrasound” or “ultrashortwave” or “inferential current” or “magnetic field” or “electromagnetic” or “light therapy” or “shockwave” or “electrostimulation” or “over-the-counter” or “medical device” or “orthotics” or “orthoses” or “orthotic device” or “insole” or “foot orthoses” or “brace” or “support” or “belt” or “corset” or “rocker shoes” or “walking stick” or “elbow crutches” or “complementary and alternative therapy” or “acupuncture” or “traditional Chinese medicine” or “electro-acupuncture” or “moxibustion” or “scalp acupuncture” or “acupoint” or “auricular therapy” or “catgut embedding” or “phytotherapeutics” or “phytotherapy” or “topical phytotherapy” or “lamp” or “salt” or “mattress” or “flooring” or “psychological intervention” or “psychological therapy” or “behavior therapy” or “multidisciplinary treatment” or “multidisciplinary biopsychosocial rehabilitation” or “workplace intervention” or “workplace adjustment” or “imaging” or “surgery”):**ti,ab,kw in Trials -(Word variations have been searched)**  #4 (“clinical trial” or “clinical study” or “comparative study” or “controlled clinical trial” or “pragmatic clinical trial” or “randomized controlled trial”):**ti,ab,kw in Trials -(Word variations have been searched)**  #5 (“China” or “Chinese”) **-(Word variations have been searched)**  #6 #1 and #3 and #4 and #5  #7 #2 and #3 and #4 and #5  #8 #6 or #7 |
| MEDLINE | #1 exp back pain/  #2 ("low back pain" or "low back ache" or "low backache" or "low back injury" or "low back disorder" or "lumbago" or "sciatica" or "spondylosis").**ti,ab,kw,kf.**  #3 (“nonpharmacological” or “advice” or “education” or “thermos therapy” or “heat” or “hot” or “cold” or “bath” or “sauna” or “fumigation” or “steaming” or “spa” or “manual therapy” or “massage” or “mobilization” or “manipulation” or “exercise” or “Qi Gong” or “Tai Chi” or “postural therapy” or “traction” or “tape” or “kinesiotape” or “patch” or “band” or “electrotherapy” or “laser therapy” or “TENS” or “PENS” or “shortwave diathermy” or “ultrasound” or “ultrashortwave” or “inferential current” or “magnetic field” or “electromagnetic” or “light therapy” or “shockwave” or “electrostimulation” or “over-the-counter” or “medical device” or “orthotics” or “orthoses” or “orthotic device” or “insole” or “foot orthoses” or “brace” or “support” or “belt” or “corset” or “rocker shoes” or “walking stick” or “elbow crutches” or “complementary and alternative therapy” or “acupuncture” or “traditional Chinese medicine” or “electro-acupuncture” or “moxibustion” or “scalp acupuncture” or “acupoint” or “auricular therapy” or “catgut embedding” or “phytotherapeutics” or “phytotherapy” or “topical phytotherapy” or “lamp” or “salt” or “mattress” or “flooring” or “psychological intervention” or “psychological therapy” or “behavior therapy” or “multidisciplinary treatment” or “multidisciplinary biopsychosocial rehabilitation” or “workplace intervention” or “workplace adjustment” or “imaging” or “surgery”).**ti,ab,kw,kf.**  #4 (“clinical trial” or “clinical study” or “comparative study” or “controlled clinical trial” or “pragmatic clinical trial” or “randomized controlled trial”).**pt.**  #5 (“China” or “Chinese”).**ti,ab,kw,kf.**  #6 #1 and #3 and #4 and #5  #7 #2 and #3 and #4 and #5  #8 #6 or #7 |
| EMBASE | #1 ‘back pain’/exp  #2 (‘low back pain’ OR ‘low back ache’ OR ‘low backache’ OR ‘low back injury’ OR ‘low back disorder’ OR ‘lumbago’ OR ‘sciatica’ OR ‘spondylosis’):**ti,ab,kw**  #3 (‘nonpharmacological’ or ‘advice’ or ‘education’ or ‘thermos therapy’ or ‘heat’ or ‘hot’ or ‘cold’ or ‘bath’ or ‘sauna’ or ‘fumigation’ or ‘steaming’ or ‘spa’ or ‘manual therapy’ or ‘massage’ or ‘mobilization’ or ‘manipulation’ or ‘exercise’ or ‘Qi Gong’ or ‘Tai Chi’ or ‘postural therapy’ or ‘traction’ or ‘tape’ or ‘kinesiotape’ or ‘patch’ or ‘band’ or ‘electrotherapy’ or ‘laser therapy’ or ‘TENS’ or ‘PENS’ or ‘shortwave diathermy’ or ‘ultrasound’ or ‘ultrashortwave’ or ‘inferential current’ or ‘magnetic field’ or ‘electromagnetic’ or ‘light therapy’ or ‘shockwave’ or ‘electrostimulation’ or ‘over-the-counter’ or ‘medical device’ or ‘orthotics’ or ‘orthoses’ or ‘orthotic device’ or ‘insole’ or ‘foot orthoses’ or ‘brace’ or ‘support’ or ‘belt’ or ‘corset’ or ‘rocker shoes’ or ‘walking stick’ or ‘elbow crutches’ or ‘complementary and alternative therapy’ or ‘acupuncture’ or ‘traditional Chinese medicine’ or ‘electro-acupuncture’ or ‘moxibustion’ or ‘scalp acupuncture’ or ‘acupoint’ or ‘auricular therapy’ or ‘catgut embedding’ or ‘phytotherapeutics’ or ‘phytotherapy’ or ‘topical phytotherapy’ or ‘lamp’ or ‘salt’ or ‘mattress’ or ‘flooring’ or ‘psychological intervention’ or ‘psychological therapy’ or ‘behavior therapy’ or ‘multidisciplinary treatment’ or ‘multidisciplinary biopsychosocial rehabilitation’ or ‘workplace intervention’ or ‘workplace adjustment’ or ‘imaging’ or ‘surgery’):**ti,ab,kw**  #4 (‘clinical trial’ or ‘clinical study’ or ‘comparative study’ or ‘controlled clinical trial’ or ‘pragmatic clinical trial’ or ‘randomized controlled trial’):**ti,ab,kw**  #5 (“China” or “Chinese”):**ti,ab,kw**  #6 #1 and #4 and #5  #7 #2 and #4 and #5  #8 #3 and #4 and #5  #9 #6 or #7 or #8 |
| CINAHL | #1 MH back pain+ in **PT** (Clinical Trial or Randomized Controlled Trial)  #2 **TI** (‘low back pain’ OR ‘low back ache’ OR ‘low backache’ OR ‘low back injury’ OR ‘low back disorder’ OR ‘lumbago’ OR ‘sciatica’ OR ‘spondylosis’) **OR AB** (‘low back pain’ OR ‘low back ache’ OR ‘low backache’ OR ‘low back injury’ OR ‘low back disorder’ OR ‘lumbago’ OR ‘sciatica’ OR ‘spondylosis’) **OR MM** (‘low back pain’ OR ‘low back ache’ OR ‘low backache’ OR ‘low back injury’ OR ‘low back disorder’ OR ‘lumbago’ OR ‘sciatica’ OR ‘spondylosis’) in **PT** (Clinical Trial or Randomized Controlled Trial)  #3 **TI** (‘nonpharmacological’ or ‘advice’ or ‘education’ or ‘thermos therapy’ or ‘heat’ or ‘hot’ or ‘cold’ or ‘bath’ or ‘sauna’ or ‘fumigation’ or ‘steaming’ or ‘spa’ or ‘manual therapy’ or ‘massage’ or ‘mobilization’ or ‘manipulation’ or ‘exercise’ or ‘Qi Gong’ or ‘Tai Chi’ or ‘postural therapy’ or ‘traction’ or ‘tape’ or ‘kinesiotape’ or ‘patch’ or ‘band’ or ‘electrotherapy’ or ‘laser therapy’ or ‘TENS’ or ‘PENS’ or ‘shortwave diathermy’ or ‘ultrasound’ or ‘ultrashortwave’ or ‘inferential current’ or ‘magnetic field’ or ‘electromagnetic’ or ‘light therapy’ or ‘shockwave’ or ‘electrostimulation’ or ‘over-the-counter’ or ‘medical device’ or ‘orthotics’ or ‘orthoses’ or ‘orthotic device’ or ‘insole’ or ‘foot orthoses’ or ‘brace’ or ‘support’ or ‘belt’ or ‘corset’ or ‘rocker shoes’ or ‘walking stick’ or ‘elbow crutches’ or ‘complementary and alternative therapy’ or ‘acupuncture’ or ‘traditional Chinese medicine’ or ‘electro-acupuncture’ or ‘moxibustion’ or ‘scalp acupuncture’ or ‘acupoint’ or ‘auricular therapy’ or ‘catgut embedding’ or ‘phytotherapeutics’ or ‘phytotherapy’ or ‘topical phytotherapy’ or ‘lamp’ or ‘salt’ or ‘mattress’ or ‘flooring’ or ‘psychological intervention’ or ‘psychological therapy’ or ‘behavior therapy’ or ‘multidisciplinary treatment’ or ‘multidisciplinary biopsychosocial rehabilitation’ or ‘workplace intervention’ or ‘workplace adjustment’ or ‘imaging’ or ‘surgery’) **OR AB** (‘nonpharmacological’ or ‘advice’ or ‘education’ or ‘thermos therapy’ or ‘heat’ or ‘hot’ or ‘cold’ or ‘bath’ or ‘sauna’ or ‘fumigation’ or ‘steaming’ or ‘spa’ or ‘manual therapy’ or ‘massage’ or ‘mobilization’ or ‘manipulation’ or ‘exercise’ or ‘Qi Gong’ or ‘Tai Chi’ or ‘postural therapy’ or ‘traction’ or ‘tape’ or ‘kinesiotape’ or ‘patch’ or ‘band’ or ‘electrotherapy’ or ‘laser therapy’ or ‘TENS’ or ‘PENS’ or ‘shortwave diathermy’ or ‘ultrasound’ or ‘ultrashortwave’ or ‘inferential current’ or ‘magnetic field’ or ‘electromagnetic’ or ‘light therapy’ or ‘shockwave’ or ‘electrostimulation’ or ‘over-the-counter’ or ‘medical device’ or ‘orthotics’ or ‘orthoses’ or ‘orthotic device’ or ‘insole’ or ‘foot orthoses’ or ‘brace’ or ‘support’ or ‘belt’ or ‘corset’ or ‘rocker shoes’ or ‘walking stick’ or ‘elbow crutches’ or ‘complementary and alternative therapy’ or ‘acupuncture’ or ‘traditional Chinese medicine’ or ‘electro-acupuncture’ or ‘moxibustion’ or ‘scalp acupuncture’ or ‘acupoint’ or ‘auricular therapy’ or ‘catgut embedding’ or ‘phytotherapeutics’ or ‘phytotherapy’ or ‘topical phytotherapy’ or ‘lamp’ or ‘salt’ or ‘mattress’ or ‘flooring’ or ‘psychological intervention’ or ‘psychological therapy’ or ‘behavior therapy’ or ‘multidisciplinary treatment’ or ‘multidisciplinary biopsychosocial rehabilitation’ or ‘workplace intervention’ or ‘workplace adjustment’ or ‘imaging’ or ‘surgery’) **OR MM** (‘nonpharmacological’ or ‘advice’ or ‘education’ or ‘thermos therapy’ or ‘heat’ or ‘hot’ or ‘cold’ or ‘bath’ or ‘sauna’ or ‘fumigation’ or ‘steaming’ or ‘spa’ or ‘manual therapy’ or ‘massage’ or ‘mobilization’ or ‘manipulation’ or ‘exercise’ or ‘Qi Gong’ or ‘Tai Chi’ or ‘postural therapy’ or ‘traction’ or ‘tape’ or ‘kinesiotape’ or ‘patch’ or ‘band’ or ‘electrotherapy’ or ‘laser therapy’ or ‘TENS’ or ‘PENS’ or ‘shortwave diathermy’ or ‘ultrasound’ or ‘ultrashortwave’ or ‘inferential current’ or ‘magnetic field’ or ‘electromagnetic’ or ‘light therapy’ or ‘shockwave’ or ‘electrostimulation’ or ‘over-the-counter’ or ‘medical device’ or ‘orthotics’ or ‘orthoses’ or ‘orthotic device’ or ‘insole’ or ‘foot orthoses’ or ‘brace’ or ‘support’ or ‘belt’ or ‘corset’ or ‘rocker shoes’ or ‘walking stick’ or ‘elbow crutches’ or ‘complementary and alternative therapy’ or ‘acupuncture’ or ‘traditional Chinese medicine’ or ‘electro-acupuncture’ or ‘moxibustion’ or ‘scalp acupuncture’ or ‘acupoint’ or ‘auricular therapy’ or ‘catgut embedding’ or ‘phytotherapeutics’ or ‘phytotherapy’ or ‘topical phytotherapy’ or ‘lamp’ or ‘salt’ or ‘mattress’ or ‘flooring’ or ‘psychological intervention’ or ‘psychological therapy’ or ‘behavior therapy’ or ‘multidisciplinary treatment’ or ‘multidisciplinary biopsychosocial rehabilitation’ or ‘workplace intervention’ or ‘workplace adjustment’ or ‘imaging’ or ‘surgery’) in **PT** (Clinical Trial or Randomized Controlled Trial)  #4 **TI** (“China” or “Chinese”) **OR AB** (“China” or “Chinese”) **OR MM** (“China” or “Chinese”) in **PT** (Clinical Trial or Randomized Controlled Trial)  #5 #1 and #3 and #4  #6 #2 and #3 and #4  #7 #5 or #6 |
| Web of Science | #1 **Title or Abstract or Keywords**: “back pain” or “back pains” or “low back pain” or “low back ache” or “low backache” or “lumbago” or “sciatica” or “spondylosis” or “lower back pain” or “lower back ache” or “low back injury” or “low back injuries” or “low back disorder” or “low back disorders”  #2 **Title or Abstract or Keywords**: “nonpharmacological” or “advice” or “education” or “thermo therapy” or “heat” or “hot” or “cold” or “bath” or “sauna” or “fumigation” or “steaming” or “spa” or “manual therapy” or “massage” or “mobilization” or “mobilisation” or “manipulation” or “exercise” or “Qi Gong” or “Tai Chi” or “postural therapy” or “traction” or “tap*” or “kinesiotape” or “patch” or “band” or “electrotherapy” or “laser therapy” or “TENS” or “PENS” or “shortwave diathermy” or “ultrasound” or “ultrashortwave” or “inferential current” or “magnetic field” or “electromagnetic” or “light therapy” or “shockwave” or “electrostimulation” or “over-the-counter” or “medical device” or “orthotics” or “orthoses” or “orthosis” or “orthotic device” or “insole” or “foot orthoses” or “foot orthosis” or “brace*” or “support” or “belt” or “corset” or “rocker shoes” or “walking stick” or “elbow crutches” or “complementary and alternative therapy” or “acupuncture” or “traditional Chinese medicine” or “electro-acupuncture” or “moxibustion” or “scalp acupuncture” or “acupoint” or “auricular therapy” or “catgut embedding” or “phytotherapeutics” or “phytotherapy” or “topical phytotherapy” or “lamp” or “salt” or “mattress” or “flooring” or “psychological intervention” or “psychological therap*” or “behavioural therapy” or “behavior therapy” or “multidisciplinary treatment” or “multidisciplinary biopsychosocial rehabilitation” or “workplace intervention” or “workplace adjustment” or “imaging” or “surgery”  #3 **Title or Abstract or Keywords**: “China” or “Chinese”  #4 #1 and #2 and #3 |
| Wanfang | #1主题topic:("中国China" or "中国人Chinese" or "中华Chinese") and 主题topic:("非药物nonpharmacological" or "心理疗法psychological intervention/psychological therap*/advice" or "教育疗法education" or "冷疗cold" or "热疗thermo therapy/heat/hot" or "泡浴bath" or "桑拿sauna" or "蒸熏fumigation" or "熏洗steaming" or "水疗spa" or "手法治疗manual therapy" or "按摩massage" or "松动术mobilization/mobilisation" or "整脊手法manipulation" or "运动exercise" or "气功Qi Gong" or "太极Tai Chi" or "体位疗法postural therapy" or "牵引traction" or "肌内效贴tap*/kinesiotape/patch/band" or "电疗法electrotherapy" or "激光laser therapy" or "经皮电神经刺激疗法TENS/ PENS" or "短波透热疗法shortwave diathermy" or "超声波ultrasound" or "超短波ultrashortwave" or "磁疗magnetic field" or "电磁场electromagnetic" or "光疗light therapy" or "冲击波shockwave" or "电刺激electrostimulation" or "康复器械over-the-counter/medical device" or "矫形器orthotics/orthoses/ orthosis/orthotic device" or "鞋垫insole" or "踝足矫形器foot orthoses/ foot orthosis" or "支具brace*/support/belt/corset" or "前跷鞋rocker shoes" or "手杖walking stick" or "肘拐杖elbow crutches" or "辅助替代治疗complementary and alternative therapy" or "针灸acupuncture" or "中医traditional Chinese medicine" or "电针electro-acupuncture" or "灸法moxibustion" or "头针scalp acupuncture" or "穴位acupoint" or "耳穴auricular therapy" or "埋线catgut embedding" or "植物疗法phytotherapeutics/phytotherapy/topical phytotherapy" or "灯照lamp" or "烤灯lamp" or "盐salt" or "床垫mattress/ flooring" or "行为疗法behavioural therapy/behavior therapy" or "生物心理社会multidisciplinary treatment/ multidisciplinary biopsychosocial rehabilitation" or "工作场所干预workplace intervention" or "影像学imaging" or "手术surgery") and 主题topic:("腰痛back pain/back pains" or "下背痛low back pain/low back ache/low backache/lumbago" or "下腰痛low back pain/low back ache/low backache/lumbago" or "坐骨神经痛sciatica" or "低背痛lower back pain/lower back ache" or "腰骶部痛spondylosis" or "下背部疼痛lower back pain/lower back ache" or "下腰损伤low back injury/low back injuries" or "腰骶损伤low back injury/low back injuries" or "腰部功能障碍low back disorder/low back disorders" or "骶髂关节紊乱low back disorder/low back disorders")  #2题名或关键词title or keywords:("中国China" or "中国人Chinese" or "中华Chinese") and题名或关键词title or keywords:("非药物nonpharmacological" or "心理疗法psychological intervention/psychological therap*/advice" or "教育疗法education" or "冷疗cold" or "热疗thermo therapy/heat/hot" or "泡浴bath" or "桑拿sauna" or "蒸熏fumigation" or "熏洗steaming" or "水疗spa" or "手法治疗manual therapy" or "按摩massage" or "松动术mobilization/mobilisation" or "整脊手法manipulation" or "运动exercise" or "气功Qi Gong" or "太极Tai Chi" or "体位疗法postural therapy" or "牵引traction" or "肌内效贴tap*/kinesiotape/patch/band" or "电疗法electrotherapy" or "激光laser therapy" or "经皮电神经刺激疗法TENS/ PENS" or "短波透热疗法shortwave diathermy" or "超声波ultrasound" or "超短波ultrashortwave" or "磁疗magnetic field" or "电磁场electromagnetic" or "光疗light therapy" or "冲击波shockwave" or "电刺激electrostimulation" or "康复器械over-the-counter/medical device" or "矫形器orthotics/orthoses/ orthosis/orthotic device" or "鞋垫insole" or "踝足矫形器foot orthoses/ foot orthosis" or "支具brace*/support/belt/corset" or "前跷鞋rocker shoes" or "手杖walking stick" or "肘拐杖elbow crutches" or "辅助替代治疗complementary and alternative therapy" or "针灸acupuncture" or "中医traditional Chinese medicine" or "电针electro-acupuncture" or "灸法moxibustion" or "头针scalp acupuncture" or "穴位acupoint" or "耳穴auricular therapy" or "埋线catgut embedding" or "植物疗法phytotherapeutics/phytotherapy/topical phytotherapy" or "灯照lamp" or "烤灯lamp" or "盐salt" or "床垫mattress/ flooring" or "行为疗法behavioural therapy/behavior therapy" or "生物心理社会multidisciplinary treatment/ multidisciplinary biopsychosocial rehabilitation" or "工作场所干预workplace intervention" or "影像学imaging" or "手术surgery") and题名或关键词title or keywords:("腰痛back pain/back pains" or "下背痛low back pain/low back ache/low backache/lumbago" or "下腰痛low back pain/low back ache/low backache/lumbago" or "坐骨神经痛sciatica" or "低背痛lower back pain/lower back ache" or "腰骶部痛spondylosis" or "下背部疼痛lower back pain/lower back ache" or "下腰损伤low back injury/low back injuries" or "腰骶损伤low back injury/low back injuries" or "腰部功能障碍low back disorder/low back disorders" or "骶髂关节紊乱low back disorder/low back disorders")  #3 摘要abstract:("中国China" or "中国人Chinese" or "中华Chinese") and摘要abstract:("非药物nonpharmacological" or "心理疗法psychological intervention/psychological therap*/advice" or "教育疗法education" or "冷疗cold" or "热疗thermo therapy/heat/hot" or "泡浴bath" or "桑拿sauna" or "蒸熏fumigation" or "熏洗steaming" or "水疗spa" or "手法治疗manual therapy" or "按摩massage" or "松动术mobilization/mobilisation" or "整脊手法manipulation" or "运动exercise" or "气功Qi Gong" or "太极Tai Chi" or "体位疗法postural therapy" or "牵引traction" or "肌内效贴tap*/kinesiotape/patch/band" or "电疗法electrotherapy" or "激光laser therapy" or "经皮电神经刺激疗法TENS/ PENS" or "短波透热疗法shortwave diathermy" or "超声波ultrasound" or "超短波ultrashortwave" or "磁疗magnetic field" or "电磁场electromagnetic" or "光疗light therapy" or "冲击波shockwave" or "电刺激electrostimulation" or "康复器械over-the-counter/medical device" or "矫形器orthotics/orthoses/ orthosis/orthotic device" or "鞋垫insole" or "踝足矫形器foot orthoses/ foot orthosis" or "支具brace*/support/belt/corset" or "前跷鞋rocker shoes" or "手杖walking stick" or "肘拐杖elbow crutches" or "辅助替代治疗complementary and alternative therapy" or "针灸acupuncture" or "中医traditional Chinese medicine" or "电针electro-acupuncture" or "灸法moxibustion" or "头针scalp acupuncture" or "穴位acupoint" or "耳穴auricular therapy" or "埋线catgut embedding" or "植物疗法phytotherapeutics/phytotherapy/topical phytotherapy" or "灯照lamp" or "烤灯lamp" or "盐salt" or "床垫mattress/ flooring" or "行为疗法behavioural therapy/behavior therapy" or "生物心理社会multidisciplinary treatment/ multidisciplinary biopsychosocial rehabilitation" or "工作场所干预workplace intervention" or "影像学imaging" or "手术surgery") and摘要abstract:("腰痛back pain/back pains" or "下背痛low back pain/low back ache/low backache/lumbago" or "下腰痛low back pain/low back ache/low backache/lumbago" or "坐骨神经痛sciatica" or "低背痛lower back pain/lower back ache" or "腰骶部痛spondylosis" or "下背部疼痛lower back pain/lower back ache" or "下腰损伤low back injury/low back injuries" or "腰骶损伤low back injury/low back injuries" or "腰部功能障碍low back disorder/low back disorders" or "骶髂关节紊乱low back disorder/low back disorders")  #4 #1 or #2 or #3 |

Note: To facilitate understanding, English translations were placed directly after their corresponding Chinese terms. The Chinese translation of "inferential current" or “interferential current lacked clarity and was replaced by TENS in Wanfang electronic database. "Over-the-counter" refers to products, such as medical device, that can be purchased without a prescription.
