# Supplementary material for: The effectiveness of non-pharmacological interventions for low back pain in China: A systematic review and network meta-analysis
Source: PLoS One. 2025 May 9;20(5):e0322929. doi: 10.1371/journal.pone.0322929 (PMC12063812; doi:10.1371/journal.pone.0322929)
Supplement: S2 Protocol — (PDF) [file pone.0322929.s002.pdf]

Effect of non-pharmacological intervention for adults with low back pain in China: A systematic review and meta-analysis

Junshi Liu, Wengkung Peng, Yingxian Jia

To enable PROSPERO to focus on COVID-19 submissions, this registration record has undergone basic automated checks for eligibility and is published exactly as submitted. PROSPERO has never provided peer review, and usual checking by the PROSPERO team does not endorse content. Therefore, automatically published records should be treated as any other PROSPERO registration. Further detail is provided here.

Citation
Junshi Liu, Wengkung Peng, Yingxian Jia. Effect of non-pharmacological intervention for adults with low back pain in China: A systematic review and meta-analysis. PROSPERO 2024 Available from
https://www.crd.york.ac.uk/prospero/display_record.php?ID=CRD42024578680

PREVIEW

REVIEW TITLE AND BASIC DETAILS

Review title

Effect of non-pharmacological intervention for adults with low back pain in China: A systematic review and meta-analysis

Review objectives

This review aimed to complete a systemtic review and meta-analysis for the effect of non-pharmacological intervention for adults with low back pain in Chia. The included trials should recruit patients over 18 years old with subtypes of low back pain who received non-pharmacological intervention in the experimental group of randomized controlled trials. The control group may take no intervention, minimal intervention, or active intervention in the trials. We aimed to extract outcome involving the post-intervention improvement on pain intensity, disability, and intervention efficacy.

SEARCHING AND SCREENING

Searches

Electronic databases of The Cochrane Library, MEDLINE, Embase, PubMed, CINAHL, and Web of Science were put into use from inception to May 14th of 2024. Only publication in English was targeted. Articles should contain trials carried out in China and patients should be the residents in China. Patients were reporting low back pain when they were reruited in the study and were provided with non-pharmacological intervention in the experimental group. Patients in the control group may take no intervention, medications, minimal intervention, or another active intervention during the entire process for comparison purpose.

Study design

The inclusion criteria: (1) randomized controlled trials having non-pharmacological intervention in the experimental group for LBP.
The exclusion criteria: (1) studies published as protocol, trial registry record, pilot trial, crossover trial, reviews, abstract, comments, short communication, discussions, thesis, conference proceedings, and retracted article.

ELIGIBILITY CRITERIA

Condition or domain being studied

Low back pain (LBP) is a well-known musculoskeletal disorder leading to disability with the highest prevalence world-wide. The clinical course of LBP can be divided into acute phase (< 6 weeks), subacute phase (6 weeks to 12 weeks), and chronic phase (> 12 weeks). The nociceptive input model pointed out the three sources for LBP. They are non-identifiable source for non-specific LBP, neurological source and specific pathology for the rest types of LBP. LBP with specific cause accounted for small portion, and non-specific LBP was most reported.

Population

The inclusion criteria: (1) subjects > 18 years old with subtypes of LBP who received non-pharmacological intervention in the experimental group of randomized controlled trials; (3) These studies were carried out in China and recruited subjects were residents in this country.
The exclusion criteria: (1) Trials used healthy subject in the control group without any method of risk minimization. (2) Low back pain was from the postoperative or postpartum pain, low back pain occurred during labor in women; (3) There are irrelevant aches from other parts of body in the experimental group, such as shoulder pain or chest pain

Intervention(s) or exposure(s)

The inclusion criteria: (1) non-pharmacological intervention in the experimental group of randomized controlled trials;
The exclusion criteria: (1) The intervention aimed for an underlying disease characterized by symptoms of low back pain as a minor problem; (2) Nerve blocks was used in the intervention

Comparator(s) or control(s)

The inclusion criteria: (1) Trials recruited LBP in the group as comparator or control group in the RCT.
The exclusion criteria: (1) Trials used healthy subject with risk of LBP in the control group without any method of risk minimization; (2) Trials only had experimental groups using intervention without a group having no intervention, sham intervention, minimal intervention, alternative intervention, or usual care as comparator

OUTCOMES TO BE ANALYSED

Main outcomes

The inclusion criteria: (1) The outcome measures had quantitative information of the effectiveness of the intervention.
The exclusion criteria: (1) The outcome assessment was only for nursing or pain management effectiveness.

Measures of effect
Intervention efficacy was calculated by risk ratio (RR) of ineffective cases ratio from the experimental group divided by the ratio from the control group. RR < 1 indicated that less ineffective cases in the experimental group compared with the control group. Standardized mean difference (SMD, Hedges' g) was applied for the analysis of pain intensity and disability. Hedges' g can be interpreted as small effect ($0.2 \le g < 0.5$), medium effect ($0.5 \le g < 0.8$), and large effect ($g > 0.8$). The post-intervention measurement was considered. When trials used 'per-protocol' effect, only patients conducted post-intervention measurement were used. The sample size remained the same pre- and post-intervention for trials using 'intention-to-treat' effect.

Additional outcomes

Not applicable

Measures of effect
Not applicable

DATA COLLECTION PROCESS

Data extraction (selection and coding)

The inclusion criteria: (1) studies included subjects over 18 years old with subtypes of low back pain who received non-pharmacological intervention in the experimental group of randomized controlled trials; (2) The outcome measures had quantitative information of the effectiveness of the intervention; (3) These studies were carried out in China and recruited subjects were residents in this country.
For eligible studies, information of authors, year of publication, study design, age, gender, subtypes of low back pains and characteristics, intervention settings (e.g., number of groups and size, number of intervention sessions and frequencies, total intervention time), outcome measurement regarding pain intensity, disability, and intervention efficacy improvement, assessment timepoint were extracted.
Information extraction was conducted by two independent researchers in a previously prepared electronic excel form, discrepancies between the researchers were reviewed and resolved by a third reviewer.

Risk of bias (quality) assessment

Cochrane Risk of Bias 2.0 (RoB2) tool was applied for all trials. This tool has five domains, including randomization process, deviations from planned interventions (effect of adhering to intervention and assignment to intervention), missing outcome data, outcome measurement, and selection of reported outcome.

PLANNED DATA SYNTHESIS

Strategy for data synthesis

The 'metagen', 'metafor', and 'meta' packages from R statistics software version 4.3.2 (The R Foundation, Vienna, Austria) were used for all statistical analyses in this study. Due to the clinical heterogeneity for the included trials, a random effects model with Restricted Maximum-Likelihood (REML) estimator was used by an assumption that different studies were estimating different but related intervention effect.

Heterogeneity was considered with Cochran Q test ($p < 0.05$) supplemented by I^2 statistic. Low level of heterogeneity was 25% < I^2 < 50%, moderate level was 50% < I^2 < 75%, and substantial level was I^2 > 75%. The level of heterogeneity was also assessed by the prediction interval which indicates the dispersion of estimated effect.

Heterogeneity analysis was carried out in random effects subgroup analysis and meta-regression analysis for categorical and continuous variables, respectively.

Analysis of subgroups or subsets

Subgroup analysis will carry out for the following variables:

Demographic variable of age was classified as adults (18 – 65 years old) and elders (> 65 years old). Low back pain was listed as the characters of pain diagnosis. Pain characters (i.e., specific low back pain, non-specific low back pain) were analyzed. Types of intervention and control methods were put together to represent the intervention setting. The method was categorized as 'minimal intervention' for using medicine, no intervention, or sham intervention, 'independent intervention' for only one type of non-pharmacological intervention, or 'combined intervention' for more than two types of non-pharmacological interventions. Intervention duration was input as the number of weeks, and they were also categorized as 'momentary' (≤ 1 week), 'long' (1 – 4 week), or 'prolonged' (> 4 week). Study quality was qualified by the overall risk-of-bias assessment.

REVIEW AFFILIATION, FUNDING AND PEER REVIEW

Review team members

- Dr Junshi Liu, Dongguan University of Technology
- Professor Wengkung Peng, Osaka University
- Dr Yingxian Jia, Department of reproductive medicine, Guizhou provincial people's hospital

Review affiliation

Dongguan University of Technology

Funding source

No funding was received from any third parties.

Named contact

Junshi Liu. Physical Education Department, Education School, Dongguan University of Technology, Daxue Rd. No. 1, Songshan Lake, Dongguan City, Guangdong Province, China.
junshi100610@gmail.com

TIMELINE OF THE REVIEW

Review timeline

Start date: 01 May 2024. End date: 01 October 2024

Date of first submission to PROSPERO

12 August 2024

Date of registration in PROSPERO

24 August 2024

AVAILABILITY OF FULL PROTOCOL

Availability of full protocol

No preview available

CURRENT REVIEW STAGE

Publication of review results
The intention is to publish the review once completed.The review will be publcished in English

Stage of the review at this submission

Review stage	Started	Completed
Pilot work	✓	✓
Formal searching/study identification	✓	✓
Screening search results against inclusion criteria	✓	✓
Data extraction or receipt of IPD	✓	✓
Risk of bias/quality assessment	✓	✓
Data synthesis	✓	✓

The study protocol has been completed

Review status
The review is completed.

ADDITIONAL INFORMATION

PROSPERO version history

- Version 1.1 published on 24 Aug 2024
- Version 1.0 published on 24 Aug 2024

Review conflict of interest
None known

Country
China

Medical Subject Headings
Adolescent; Adult; Control Groups; Disabled Persons; Humans; Low Back Pain; Pain Measurement; Randomized Controlled Trials as Topic

Revision note
After reviewing process in the journal of PloS One, the title has been changed to "The effectiveness of non-pharmacological interventions for low back pain in China: A systematic review and network meta-analysis". Relevant methodology regarding data curation and analysis assessment has been updated and can be found in the accepted manuscript. These changes have not changed the purpose and objective of this study. It still aligns with the previous registration in PROSPERO.

Disclaimer
The content of this record displays the information provided by the review team. PROSPERO does not peer review registration records or endorse their content.

PROSPERO accepts and posts the information provided in good faith; responsibility for record content rests with the review team. The owner of this record has affirmed that the information provided is truthful and that they understand that deliberate provision of inaccurate information may be construed as scientific misconduct.

PROSPERO does not accept any liability for the content provided in this record or for its use. Readers use the information provided in this record at their own risk.

Any enquiries about the record should be referred to the named review contact
