# Supplementary material for: The effectiveness of non-pharmacological interventions for low back pain in China: A systematic review and network meta-analysis
Source: PLoS One. 2025 May 9;20(5):e0322929. doi: 10.1371/journal.pone.0322929 (PMC12063812; doi:10.1371/journal.pone.0322929)
Supplement: S3 Table — Adr, adrenaline; ASLR, angle of straight leg raising; β-EP, beta-endorphins; bid, twice a day; BMI, body mass index; BP, blood pressure; BPI-SF, brief pain inventory-short form; BSS, biodex stability system; CLBP, chronic low back pain; CNSLBP, chronic non-specific low back pain; CSE, core stability exercise; C-SF-MPQ, Chinese short-form of McGill pain questionnaire; C-SFODI, simplified Chinese version of the Oswestry Disability Index; CTR, control group; d, day; DAL, daily activity level; dep, dependent; DLBP, discogenic low back pain; DLSS, degenerative lumbar spinal stenosis; DMS, deep muscle stimulation; EA, electroacupuncture; EAS, electronic acupuncture shoes; exerc, exercise; ESWT, extracorporeal shock wave therapy; EXP, experimental group; F, female; FABQ, fear avoidance beliefs questionnaire; FAI, Frenchay activity index; 5-HT, 5-hydroxytryptamine; GAD-7, 7-item generalized anxiety disorder scale; GQOL-74, generic quality of life inventory-74; HADS, hospital anxiety and depression scale; H-MRS, hydrogen-1 magnetic resonance spectroscopy; HR, heart rate; HRV, heart rate variability; IL, interleukin; IMS, isometric muscle strength; int, intervention; JOA, Japanese Orthopaedic Association score; LBP, low back pain; LDD, lumbosacral disc degeneration; LDE, lumbar dynamic exercise; LDH, lumbar disc herniation; LPL, low power laser; M, male; m, mean; mALBPDS, modified Aberdeen low back pain disability scale; MCID, minimal clinical important difference; MCS, mental component summary; MET, motivational enhancement therapy; MFI, multidimensional fatigue inventory; min, minute; mJOA, modified Japanese Orthopaedic Association score; mo, month; mODI, modified Oswestry disability index; NA, not applicable; NE, norepinephrine; NRS, numeric rating scale; NSAIDs, nonsteroidal anti-inflammatory drugs; NSLBP, non-specific low back pain; OA-QALQ, osteoarthritis quality of life questionnaire; ODI, Oswestry disability index; PASS, pain anxiety symptom scale; PCS*, pain catastro [file pone.0322929.s003.docx]

| **Authors & year** | **Age (m ± std)/types of LBP and symptoms** | **Intervention settings** | **Outcome measurement** | **Assessment time point** |
| --- | --- | --- | --- | --- |
| Gan, et al. (2024)  [26] | CTR: 45.7 ± 2.2  EXP: 45.4 ± 2.8  / LBP: LDH | CTR (M = 41, F = 22): acupuncture [30-min qd]  EXP (M = 46, F = 26): CTR [20-min qd] + acupoint massage [5 – 10-min qd]  int. duration: 2-w  follow-up time: none  medications: none | VAS  JOA  overall efficacy (symptoms improvement)  time of symptoms disappearance  GQOL-74  clinical satisfaction | pre- and post-int. |
|  | Acupuncture: It is a cornerstone of Traditional Chinese Medicine (TCM), which posits that vital energy, known as 'qi,' flows through pathways within the body called meridians. Pain arises when the flow of qi in these meridians is obstructed. Acupuncture involves inserting sterile needles into specific points (acupoints) on the body to stimulate qi and alleviate pain. These acupoints, identified and utilized by TCM experts over centuries, are strategically located on the body, including the lumbar region, buttocks, and legs for low back pain treatment.  Massage: In TCM, massage shares a similar therapeutic principle with acupuncture. Both aim to regulate the flow of 'qi' within the body. However, instead of using needles, massage employs manual pressure applied to specific acupoints to stimulate qi and alleviate pain. | | | |
| He, et al. (2024)  [27] | CTR: 24.89 ± 4.03  EXP: 26.43 ± 5.07  / CNSLBP: VAS 3 – 7 pts | CTR (M = 62): medications [7-d]  EXP (M = 68): cupping and scraping [2 sess. on 1st-d & 4th-d]  int. duration: 7-d  follow-up time: none  medications: diclofenac sodium [100 mg/d] + capsaicin plaster [two patches] | VAS  JOA  TCMS  overall efficacy (% improvement on VAS) | pre-int., 4th-d of int., post-int. |
|  | Cupping and scraping: Cupping and scraping, like massage, operate based on similar therapeutic principles in TCM. While massage utilizes manual pressure, cupping techniques, including static and sliding cupping, generate varying degrees of pressure by manipulating negative pressure within cups. Although these methods are often attributed a thermogenic effect, we believe this effect is likely transient and incomparable to the sustained heat produced by moxibustion. | | | |
| Shi, et al. (2024)  [38] | CTR: 49.82 ± 4.11  EXP: 50.09 ± 4.32  / LBP: LDH | CTR (M = 22, F = 38): usual care + LDE [1×/d]  EXP (M = 24, F = 36): usual care + CSE [1×/d]  int. duration: 2-mo  follow-up time: none  medications: none | VAS  mJOA  WHOQOL-BREF  lumbar ROM | pre-int., 1st-mo of int., post-int. |
|  | LDE (lumbar dynamic exercise): These exercises comprise a series of dynamic joint movements characterized by repetitive actions within a specific range of motion for a selected joint. Focusing on low back pain, these exercises emphasize hip and spinal joint movements, particularly those involving joints flexion and extension. To broaden the scope of these exercises, we also consider aquatic exercises, swimming, jogging, and backward walking as forms of lumbar dynamic exercise. However, regular walking, lacking a specific training objective, was categorized as usual care.  CSE (core stability exercise): While sharing a repetitive nature with lumbar dynamic exercises, these exercises involve a series of static joint movements characterized by sustained stabilization of a selected joint. Unlike dynamic exercises, these movements emphasize isometric contractions of muscles surrounding the hip and lumbar joints, holding the joint in a stable position for an extended duration compared to the movement time in dynamic exercises. | | | |
| Zheng, et al. (2024)  [49] | CTR: 38.2 ± 10.5  EXP: 32.2 ± 8.6  / CNSLBP: > 12-w RMDQ > 4 pts | CTR (M = 6, F = 19): CSE [30 – 40-min 4×/w]  EXP (M = 4, F = 23): CTR + SCT [4 sess.]  int. duration: 4-w  follow-up time: 48-w  medications: none | NRS  RMDQ  PHQ-9  GAD-7  PCS^*^  PSEQ  SCS | pre-int., 2nd-w of int., post-int., 12-, 48-w post-int. |
|  | SCT: This therapy employs psychosocial interventions to address the mental aspects of low back pain. We believe that educational therapy, motivational enhancement therapy also falls within this category of intervention. In meta-analysis, they are categorized as ‘mental related therapy’. | | | |
| Zuo, et al. (2024)  [60] | EXP1: 40.8 ± 8.1  EXP2: 36.6 ± 7.3  EXP3: 37.5 ± 8.2  / CNSLBP: > 12-w  VAS > 2 pts | EXP1 (n = 19): CSE [~45-min 5×/w]  EXP2 (n = 15): interferential current [~30-min 5×/w]  EXP3 (n = 19): EXP1 [3×/w 6-w, 2×/w 6-w] + EXP2 [2×/w 6-w, 3×/w 6-w]  int. duration: 12-w  follow-up time: none  medications: none | VAS  ODI  SF-12  trunk muscle strength, endurance, ROM | pre- and post-int. |
|  | Inferential or interferential current therapy was categorized as TENS. | | | |
| Cheng, et al. (2023)  [71] | CTR: 41.23 ± 11.04 EXP1: 38.73 ± 10.39  EXP2: 40.17 ± 10.67  EXP3: 39.96 ± 10.42  / CNSLBP: > 12-w; | CTR (M = 15, F = 15): no int.  EXP1 (M = 16, F = 14): EXP2 + EXP3  EXP2 (M = 14, F = 16): intermittent wave EA [15 - 20-min 5×/w]  EXP3 (M = 18, F = 12): CSE [3×/w]  int. duration: 2-w  follow-up time: none  medications: none | VAS  ODI  overall efficacy (symptoms improvement) | pre- and post-int. |
|  | Electroacupuncture which is acupuncture combined with inferential current was categorized as PENS. | | | |
| Ju (2023)  [78] | CTR: 46.32 ± 1.28  EXP: 46.26 ± 1.31  /LBP: LDH | CTR (M = 14, F = 16): bilateral intervertebral cage implant fusion  EXP (M = 13, F = 17): unilateral intervertebral cage implant fusion  int. duration: none  follow-up time: 3-mo  medications: none | blood loss during surgery  surgery duration  length of stay  VAS  PSQI  SF-36 | pre-int., 1st-, 3rd-mo post-int. |
| Li, et al. (2023)  [79] | CTR: 50.23 ± 6.94  EXP: 50.41 ± 6.88  / LBP: LDH | CTR (M = 30, F = 20): acupuncture [50-min qd]  EXP (M = 31, F = 19): acupuncture with different needle inserting technique [50-min qd]  int. duration: 10-d  follow-up time: none  medications: none | VAS  RMDQ  JOA  overall efficacy (% improvement on VAS) | pre- and post-int. |
|  | Acupuncture with different needle inserting technique: For this study, we grouped several acupuncture-related therapies under the category of "acupuncture plus." These therapies may involve variations in needle inserting techniques, target muscle trigger points instead of acupoints, utilize auricular, hand, or foot acupoints for treating low back pain, or employ intensive acupuncture with many needles, or acupuncture sessions at specific time in one day. | | | |
| Liu, et al. (2023)  [80] | CTR: 26  EXP1: 27  EXP2: 24  / CNSLBP: > 12-w  VAS > 3 pts  one episode past 2-w | CTR (M = 4, F = 7): no int.  EXP1 (M = 4, F = 7): acupuncture [30-min 3×/w]  EXP2 (M = 5, F = 6): trigger point acupuncture [10-min 3×/w]  int. duration: 4-w  follow-up time: 4-w  medications: none | VAS  ODI  3D gait analysis  lumbar muscles sEMG during the gait  lumbar ROM | pre- and post-int., 4-w post-int. |
| Qiao, et al. (2023)  [81] | CTR: 41.13 ± 15.11  EXP: 46.47 ± 13.27  / NSLBP | CTR (M = 15, F = 16): CSE [30-min 5×/w]  EXP (M = 16, F = 14): CTR + ESWT [1×/w]  int. duration: 4-w  follow-up time: none  medications: none | VAS  ODI  RMDQ  TNF-α  IL-1β  Adr  NE  pelvic blood flow | pre- and post-int. |
|  | ESWT: It utilizes acoustic energy to stimulate the body's natural healing mechanisms. This stimulation enhances blood circulation to the treatment area and concurrently reduces inflammation and swelling. | | | |
| Wang, et al. (2023)  [28] | CTR: 25  EXP: 26  / CNSLBP: > 3-mo  VAS > 3 pts | CTR (M = 5, F = 12): CSE [30-min/sess. 3×/w]  EXP (M = 5, F = 12): progressive postural control exerc. [3 stages split into 2-w, 3-w, 3-w 35-min/sess.]  int. duration: 8-w  follow-up time: 6-mo  medications: none | VAS  ODI  RMDQ  muscle thickness  muscle contractility  postural control | pre- and post-int., 6-mo post-int. |
|  | Progressive postural control exercise: It comprises of static and dynamic exercises. Training content is modified throughout the program, while the intensity and volume of the exercises are systematically increased across various stages. | | | |
| Yang, et al. (2023a)  [29] | CTR: 31.78 ± 2.59  EXP: 31.24 ± 3.82  / CNSLBP: NRS < 6 | CTR (M = 13, F = 14): walking exerc. [30-min/sess. 5×/w]  EXP (M = 14, F = 15): Baduanjin [30-min/sess. 5×/w]  int. duration: 4-w  follow-up time: none  medications: none | NRS  ODI  sEMG at lumbar flexion and extension | pre- and post-int. |
|  | Baduanjin: This exercise regimen evolved from Traditional Chinese Exercises (TCE), a category that encompasses martial arts. It incorporates a series of dynamic joint movements, demanding mindful execution from practitioners. Successful performance requires a combination of balance, coordination, strength, flexibility, and pulmonary fitness. Notable examples of this exercise type include Tai Chi, Wuqinxi, and Yijinjin.  Regular walking, normal daily activities, and similar interventions were categorized as "usual care." | | | |
| Yang, et al. (2023b)  [30] | CTR: 36.53 ± 8.17  EXP: 36.71 ± 8.91  / NSLBP: VAS > 3 pts | CTR (n = 38): sham intervention [2×/w]  EXP (n = 38): low-level laser acupuncture [2×/w] + auricular acupoint acupuncture [2×/w]  int. duration: 4-w  follow-up time: none  medications: irregularly painkiller + muscle relaxant | VAS  RMDQ  BPI-SF | pre-int., 2nd-w of int., post-int. |
|  | Laser acupuncture: It utilizes light energy for therapeutic purposes. This therapy is classified as light therapy rather than 'acupuncture plus'. | | | |
| Chen, et al. (2022)  [34] | EXP1: 36.52 ± 13.35  EXP2: 35.74 ± 10.62  / CNSLBP: > 3-mo  VAS ≥ 5 pts | EXP1 (M = 13, F = 20): low intensity ESWT [3×/w]  EXP2 (M = 11, F = 24): medium intensity ESWT [1×/w]  int. duration: 2-w  follow-up time: 2.5-mo  medications: as needed for aggravated symptoms | VAS  ODI  HADS | pre- and post-int., 2-w, 4-w, and 2.5-mo post-int. |
|  | This study used medium-intensity moxibustion as the standard treatment. Treatments with higher intensity or dosage were classified as "moxibustion plus," while those with lower intensity or dosage were classified as "moxibustion minus." | | | |
| Huo, et al. (2022)  [35] | CTR: 38.2 ± 6.5  EXP: 40.4 ± 5.5  / CLBP: > 3-mo  VAS ≥ 3 pts  C-SFODI ≥ 20% | CTR (M = 6, F = 7): no int.  EXP (M = 6, F = 7): acupoint massage [2×/w]  int. duration: 20-d  follow-up time: none  medications: none | VAS  C-SFODI  H-MRS | pre-int., 1st, 3rd, 6th of int. |
| Li (2022)  [31] | CTR: 38.8 ± 4.2  EXP: 39.2 ± 3.9  / NSLBP | CTR (M = 22, F = 18): acupoint massage [20-min qd 5-d in a row 2-d rest]  EXP (M = 23, F = 17): CTR + CSE [qd]  int. duration: 4-w  follow-up time: 6-mo  medications: none | VAS  ODI  JOA  overall efficacy (symptoms improvement) | pre- and post-int., 6-mo post-int. |
| Li, et al. (2022)  [32] | CTR: 46.39 ± 12.34  EXP: 47.01 ± 11.57  / LBP: > 12-w; LDD  VAS ≥ 5 pts | CTR (M = 31, F = 46): acupuncture [25-min 3×/w]  EXP (M = 32, F = 50): silver needle warm acupuncture [25-min 3×/w]  int. duration: 3-w  follow-up time: 25-w  medications: none | VAS  mODI  JOA | pre-int., 1-w, 13-w, and 25-w post-int. |
|  | In this study, warm acupuncture was administered using thermal equipment, and thus, categorized as 'acupuncture + thermo'. The thermos therapy can also be seen as using infrared ray, paraffino, and magnetic heat. | | | |
| Peng, et al. (2022)  [33] | CTR: 30.4 ± 11.8  EXP: 31.7 ± 11.3  / CLBP: > 3-mo  NRS ≥ 3 pts | CTR (M = 24, F = 33): inferential current + infrared ray thermal therapy [60-min 2×/w]  EXP (M = 30, F = 26): aquatic exerc. [60-min 2×/w]  int. duration: 3-mo  follow-up time: 9-mo  medications: none | NRS  RMDQ  SF-36  SAS  SDS  PSQI  PASS  TSK  FABQ  MCID in pain and function  global perceived effect  adverse events | pre- and post-int., 3-mo, and 9-mo post-int. |
| Wang, et al. (2022)  [36] | CTR: 42.39 ± 8.10  EXP: 43.56 ± 7.94  / NSLBP | CTR (M = 33, F = 37): acupuncture [30-min 5×/w]  EXP (M = 65, F = 75): intensive acupuncture [2×/w]  int. duration: 4-w  follow-up time: none  medications: none | VAS  JOA  overall efficacy (symptoms improvement) | pre-int. 2nd-w of int., post-int. |
| Zheng, et al. (2022)  [37] | CTR: 34.9 ± 14.5  EXP: 34.0 ± 14.4  / CNSLBP: > 12-w | CTR (M = 6, F = 14): LDE [20 – 45-min 3×/w]  EXP (M = 8, F = 12): CTR + education [1×/w]  int. duration: 6-w  follow-up time: 12-w  medications: none | NRS  RMDQ  SF-36  GAD-7  SDS | pre- and post-int., 12-w post-int. |
| Zhou, et al. (2022)  [39] | CTR: 51.77 ± 10.32  EXP: 44.36 ± 10.44  / LBP: LDH; > 3-mo VAS > 30 mm | CTR (M = 58, F = 71): acupoint massage [20-min 3×/w]  EXP (M = 64, F =66): CTR + TCE [3×/w]  int. duration: 6-w  follow-up time: none  medications: less than 3 times pharmacological intervention were allowed in CTR (n = 16) and EXP (n = 12) | VAS  ODI  SF-MPQ  gait performance  spinal joint angle | pre- and post-int. |
| Guo, et al. (2021)  [40] | EXP1: 34.9 ± 8.7  EXP2: 36.0 ± 11.2  EXP3: 36.5 ± 10.8  / CNSLBP > 3-mo | EXP1 (M = 25, F = 22): ESWT [1×/w] + CSE [20-min 2×/w]  EXP2 (M = 23, F = 25): medications + CSE [20-min 2×/w]  EXP3 (M = 17, F = 28): EXP1 + EXP2  int. duration: 4-w  follow-up time: 8-w  medications: celecoxib + eperisone | NRS  ODI  PSEQ  PHQ-9 | pre-int., 1st-w, 2nd-w, and 3rd-w of int., post-int., 8-w post-int. |
| Hao (2021)  [41] | CTR: 37.97 ± 18.06  EXP: 38.42 ± 20.54  / LBP: LDH | CTR (M = 27, F = 23): medications + traction [qod]  EXP (M = 25, F = 25): CTR + paraffinotherapy [30-min/time bid]  int. duration: 14-d  follow-up time: 1-w  medications: glucose + saliva injection, mannitol + dexamethasone injection, chlorzoxazone, vitamin B1 | VAS  mJOA  lumbodorsal ROM  IMS  WHOQOL-BREF  overall efficacy (symptoms improvement) | pre- and post-int. |
|  | Traction: A technique that involves gently pulling on a body part, such as the lumbar spine for low back pain. | | | |
| Hu, et al. (2021)  [42] | CTR: 48.9 ± 14.3  EXP: 50.8 ± 15.3  / CNSLBP: > 3-mo | CTR (M = 24, F = 26): usual care + medications [no information for prescription details]  EXP (M = 22, F = 28): CTR + intensive acupuncture [only one time]  int. duration: 2-w  follow-up time: 6-mo  medications: NSAIDs + sodium aescinate | NRS  ODI  SF-12  bioelectricity score  HRV | pre- and post-int., 1-mo, 2-mo, 3-mo, and 6-mo post-int. |
| Li & Zhou (2021)  [43] | EXP1: 50.32 ± 9.04  EXP2: 47.77 ± 13.90  EXP3: 50.32 ± 9.04  / LBP: LDH | EXP1 (M = 14, F = 21): continuous wave EA [20-min qd]  EXP2 (M = 13, F = 22): intermittent wave EA [20-min qd]  EXP3 (M = 13, F = 21): disperse-dense wave EA [20-min qd]  int. duration: 10-d  follow-up time: none  medications: none | VAS  JOA  overall efficacy (symptoms improvement) | pre- and post-int. |
|  | This study used continuous-wave PENS as the standard treatment. PENS treatments with lower intensity, lower dosage, or an intermittent wave pattern were categorized as "PENS minus." Conversely, treatments with higher intensity, higher dosage, or a disperse-dense wave pattern were categorized as "PENS plus." | | | |
| Lu, et al. (2021a)  [44] | CTR: 55.1 ± 5.8  EXP: 54.6 ± 5.4  / LBP: LDH | CTR (M = 20, F = 30): acupuncture [30-min qd]  EXP (M = 23, F = 27): CTR + moxibustion [15-min qd]  int. duration: 20-d  follow-up time: none  medications: none | VAS  ODI  JOA  IL-6  TNF-α  overall efficacy (symptoms improvement) | pre- and post-int. |
|  | Moxibustion: It shares the same fundamental principle as acupuncture to regulate the flow of 'qi'. However, moxibustion involves burning dried mugwort (moxa) near the skin to generate a tolerable burning sensation, which is believed to alleviate symptoms. | | | |
| Lu, et al. (2021b)  [45] | CTR: 40.23 ± 14.77  EXP: 38.43 ± 15.02  / NSLBP | CTR (M = 33, F = 9): inferential current + magnetic heat [6×/w]  EXP (M = 35, F = 8): CTR + kinesiotape [q2d] + DMS [6×/w]  int. duration: 2-w  follow-up time: none  medications: none | VAS  RMDQ | pre-int., 1st-w of int., post-int. |
| Ma, et al. (2021)  [46] | CTR: 49.23 ± 13.28  EXP: 47.67 ± 15.40  / CNSLBP: > 3-mo  VAS ≥ 3 pts | CTR (M = 11, F = 19): massage [3×/w qod 2-w, 2×/w q2d 2-w]  EXP (M = 15, F = 15): trigger point acupuncture [3×/w qod 2-w, 2×/w q2d 2-w]  int. duration: 4-w  follow-up time: 12-m  medications: none | VAS  ODI  JOA  SF-36 | pre- and post-int., 3-mo, and 12-mo post-int. |
| Yao, et al. (2020)  [47] | CTR: 54 ± 14  EXP: 53 ± 16  / CNSLBP: > 12-w | CTR (M = 8, F = 28): LDE [1-hr/sess. 4×/w]  EXP (M = 6, F = 30): TCE [1-hr/sess. 4×/w]  int. duration: 24-w  follow-up time: none  medications: none | VAS  SF-MPQ  PPI  SF-36  PSQI  PCS^**^  MCS  trunk muscle strength | pre-int., 12th-w of int., post-int. |
| Yeh, et al. (2020)  [48] | CTR: 41.1 ± 8.1  EXP: 45.7 ± 10.2  / CLBP: > 12-w | CTR (M = 15, F = 19): sham EAS [1-hr 3×/w] + medications  EXP (M = 10, F = 28): EAS [1-hr 3×/w] + placebo NSAIDs  int. duration: 6-w  follow-up time: 2-w  medications: NSAIDs | VAS  RMDQ  pain remission time  lumbar ROM  SF-36 | pre- and post-int., 2-w post-int. |
| Hu, et al. (2019)  [82] | CTR: 40.32 ± 7.92  EXP1: 38.32 ± 6.71  EXP2: 40.19 ± 5.16  / LBP: LDH | CTR (M = 13, F = 18): medications [3×/d]  EXP1 (M = 13, F = 15): acupuncture [30-min qd 10-d in a row 2-d rest]  EXP2 (M = 16, F = 15): wide-spread heat moxibustion [30-min 1×/w]  int. duration: 1-mo  follow-up time: none  medications: diclofenac sodium [75 mg/d] | ODI  overall efficacy (symptoms improvement)  yang deficiency scale | pre- and post-int. |
| Li, Yan & Huang (2019)  [50] | CTR: 37.5 ± 6.1  EXP: 37.8 ± 6.3  / DLBP | CTR (M = 40, F = 32): traction [60-min bid 4-6 h interval] + massage [30-min qd 6-d in a row 1-d rest]  EXP (M = 43, F = 33): wide-spread heat moxibustion [qd 6-d in a row 1-d rest]  int. duration: 3-w  follow-up time: none  medications: none | VAS  ODI  SF-36  IL-2  IL-10  TNF-α  overall efficacy (symptoms improvement) | pre- and post-int. |
|  | In this review, "widespread heat moxibustion" and "medium length moxibustion" were both classified under the category of "moxibustion". The rest method by the difference of intensity or dosage was labeled as “moxibustion minus” or “moxibustion plus” | | | |
| Luo, et al. (2019)  [51] | CTR: 37 ± 9  EXP1: 39 ± 9  EXP2: 36 ± 10  / CLBP: > 3-mo | CTR (M = 39, F = 9): usual care [7-w] + medications  EXP1 (M = 43, F = 11): hand-ear acupuncture (hand point: [1×/d qod 4-w, 2×/w 3-w] + auricular point: [7-d in a row, 1× q4d 7-w]  EXP2 (M = 38, F = 12): acupuncture [35-min 1×/d qod 4-w; 2×/w 3-w]  int. duration: 7-w  follow-up time: none  medications: NSAIDs | VAS  RMDQ  SF-36  overall efficacy (symptoms improvement) | pre-int., 2-m, and 6-m post-int. |
| Zou, et al. (2019)  [52] | CTR: 60.67 ± 2.58  EXP1: 58.40 ± 5.08  EXP2: 58.13 ± 5.38  / NSLBP: VAS < 8 pts | CTR (M = 3, F = 10): normal DAL  EXP1 (M = 4, F = 11): CSE [60-min 3×/w]  EXP2 (M = 4, F = 11): TCE [60-min 3×/w]  int. duration: 12-w  follow-up time: none  medications: none | VAS  dynamic strength at knee, ankle | pre- and post-int. |
| Wang, et al. (2018a)  [53] | CTR: 43.4 ± 13.8  EXP: 43.8 ± 12.7  / LBP: LDH; | CTR (M = 24, F = 26): traction + usual care  EXP (M = 22, F = 28): CTR + inferential current [30-min/time 1×/d]  int. duration: 4-w  follow-up time: none  medications: none | VAS  overall efficacy (symptoms improvement)  PRI  PPI  peripheral ROS | pre- and post-int. |
| Wang, et al. (2018b)  [54] | CTR: 33.70 ± 8.22  EXP: 34.70 ± 11.99  / CNSLBP | CTR (M = 13, F = 17): CSE [3×/w qod]  EXP (M = 11, F = 19): CTR + lumbar manipulation [20-min 3×/w qod]  int. duration: 4-w  follow-up time: none  medications: none | VAS  ODI  lumbar dynamic strength | pre-int., 1st-w, 2nd-w, 3rd-w, 4th-w of int., 1-mo, 3-mo post-int. |
|  | Manipulation: It involves a high-velocity, low-amplitude thrust to a specific joint. | | | |
| Bi, et al. (2017)  [55] | CTR: 28.67 ± 19.45  EXP: 30.15 ± 18.46  / DLBP | CTR (M = 26, F = 19): acupuncture [30-min]  EXP (M = 28, F = 17): CTR + auricular acupuncture [3×/d, q3d, alternating ears]  int. duration: one time  follow-up time: none  medications: none | McGill rating  JOA  ODI  5-HT  β-EP | pre- and post-int. |
| Xiao, Shen, & Bai (2017)  [56] | CTR: NA  EXP: NA  / NSLBP  VAS ≥ 3 pts | CTR (n = 10): no int.  EXP (n = 15): acupoint massage using foot [3×/w]  int. duration: 4-w  follow-up time: none  medications: none | VAS  lumbar endurance  single leg stance  lumbar isometric strength | pre- and post-int. |
| Wang, et al. (2017)  [57] | CTR: 60.7 ± 6.9  EXP: 62.8 ± 7.5  / DLSS | CTR (M = 27, F = 20): traction + inferential current [3×/w]  EXP (M = 25, F = 22): tendon knife [1×/w]  int. duration: 14-d  follow-up time: 6-mo  medications: none | VAS  JOA  overall efficacy ((% improvement on JOA) | pre- and post-int., 1-mo, 6-mo post-int. |
|  | Tendon knife: In Western contexts, it is often referred to as tendon scraper. While bearing some resemblance to cupping, this technique utilizes a knife-like tool to target and address muscle trigger points or fascial restrictions. | | | |
| Tang, et al. (2016)  [58] | CTR: 43.6 ± 6.4  EXP: 41.7 ± 5.6  / low grade lumbar degenerative instability LBP | CTR (M = 28, F = 13): acupoint massage [40-min qd]  EXP (M = 31, F = 10): CTR + CSE [qd]  int. duration: 6-w  follow-up time: 1-yr  medications: none | VAS  JOA | pre-int., 2nd-w of int., post-int. |
| Xu, et al. (2015)  [59] | CTR: 41.9 ± 4.8  EXP1: 42.6 ± 8.5  EXP2: 44.2 ± 5.6  EXP3: 43.4 ± 6.3  / LBP: ≥ 6-mo  VAS: ≥ 4 pts | CTR (M = 4, F = 5): acupuncture [30-min 5×/w]  EXP1 (M = 3, F = 5): short heating time length moxibustion [15-min 5×/w]  EXP2 (M = 4, F = 4): moderate heating time length moxibustion [30-min 5×/w]  EXP3 (M = 4, F = 4): long heating time length moxibustion [60-min 5×/w]  int. duration: 2-w  follow-up time: none  medications: none | VAS  RMDQ | pre- and post-int. |
| Ye, et al. (2015)  [61] | CTR: 24.18 ± 3.60  EXP: 23.64 ± 3.53 / LBP: LDH | CTR (n = 33): LPL [qd 7× 1st-w] + LDE [3×/w]  EXP (n = 30): LPL [qd 7× 1st-w] + progressive postural control exerc. [3×/w]  int. duration: 12-w  follow-up time: 1-yr  medications: none | VAS  ODI | pre-int., 1st-w of int., post-int., 1-yr post-int. |
| Zhang, et al. (2015)  [62] | CTR: 51.62 ± 4.03  EXP: 48.71 ± 3.89  / NSLBP | CTR (M = 31, F = 15): massage [40-min qd]  EXP (M = 29, F = 17): CTR + CSE [qd]  int. duration: 8-w  follow-up time: none  medications: none | VAS  ODI  recurrence rate | pre-int., 2nd-w of int., post-int., 1-yr post-int. |
| Bahrami-Taghanaki, et al. (2014)  [65] | CTR: 44.4 ± 12.0  EXP: 45.7 ± 11.3  / CLBP: > 3-mo  VAS: ≥ 50 mm | CTR (M = 10, F = 20): acupuncture [20-min qod 3×/w]  EXP (M = 8, F = 22): CTR + time acupuncture [20-min qod 3×/w]  int. duration: sess. dep. on subject satisfaction  follow-up time: 12-w  medications: none | VAS  therapy sess.  absence days  pain relapses | pre-int., 1st int., post-int., 4-w, and 12-w post-int. |
| Chen, et al. (2014)  [83] | EXP1: 46.6 ± 10.5  EXP2: 47.3 ± 11.2  EXP3: 45.5 ± 10.6  / LBP: LDH  mJOA ≥ 10 | EXP1 (M = 71, F = 81): medications + acupuncture [30-min qd]  EXP2 (M = 72, F = 80): medium length moxibustion [45 min tid 4-d in a row, qd 10-d in a row]  EXP3 (M = 74, F = 78): heat-sensitive moxibustion [30 – 60 min tid 4-d in a row, qd 10-d in a row]  int. duration: 14-d  follow-up time: 6-mo  medications: mannitol + Voltaren | mJOA | pre- and post-int., 6-mo post-int. |
|  | Heat-sensitive moxibustion is categorized into “moxibustion plus”. | | | |
| Hsieh & Lee (2014)  [64] | CTR: 58.5 ± 10.6  EXP: 60.1 ± 14.2  / CNSLBP: > 12-w | CTR (M = 8, F = 19): inactive light therapy [3×/w]  EXP (M = 14, F = 19): active light therapy [3×/w]  int. duration: 2-w  follow-up time: none  medications: none | VAS  ODI  MFI  FABQ  BSS  FAI  OA-QALQ | pre- and post-int. |
| Zhang, et al. (2014)  [63] | CTR: 23.04 ± 2.24  EXP: 22.29 ± 2.85  / CLBP: ≥ 3-mo  VAS ≤ 8 | CTR (M = 16, F = 11): LDE [40-min 3×/w]  EXP (M = 18, F = 9): CTR + education [40-min 1×/w]  int. duration: 12-w  follow-up time: none  medications: none | VAS  ODI  muscle static/dynamic endurance  SF-36 | pre- and post-int. |
| Feng, et al. (2013)  [67] | CTR: 38.2 ± 5.4  EXP: 38.5 ± 5.4  / LBP: LDH | CTR (M = 27, F =19): hot fomentation using Chinese medicine + bed rest  EXP (M = 28, F = 20): CTR + Feng’s spinal manipulation [2× or 3×/w]  int. duration: 3-w  follow-up time: none  medications: none | VAS  JOA  anatomical diameters  ASLR | pre- and post-int. |
| Wu, et al. (2013)  [66] | CTR: 38.1 ± 5.2  CTR1: 37.5 ± 5.5  CTR2: 37.2 ± 5.6  CTR3: 38.2 ± 5.8  EXP: 37.5 ± 5.2  / CNSLBP: 1 – 5 yr  VAS > 40 mm | CTR (M = 28, F = 19): usual care  CTR1 (M = 21, F = 17): CTR + swimming training [qd 5×/w]  CTR2 (M = 28, F = 19): CTR + jogging training [qd 5×/w]  CTR3 (M = 26, F = 21): CTR + backward walking training [qd 5×/w]  EXP (M = 86, F = 55): CTR + tai chi training [qd 5×/w]  int. duration: 6-mo  follow-up time: none  medications: none | VAS  BMI  HR  BP  Sleep | pre-int., 3rd-mo of int., post-int. |
| Kong, et al. (2012)  [68] | CTR: 19.95 ± 3.57  EXP: 21.18 ± 3.77  / NSLBP | CTR (M = 28, F = 27): massage + placebo ointment [30-min 2×/w]  EXP (M = 29, F = 26): massage + herbal ointment [30-min 2×/w]  int. duration: 4-w  follow-up time: 3-mo  medications: none | VAS  C-SF-MPQ  muscle stiffness | pre-int., 1st int., post-int., 1-mo, and 3-mo post-int. |
| Lin, et al. (2012)  [69] | CTR: 64.65 ± 13.57  EXP: 63.35 ± 11.23  / CLBP: > 3-mo | CTR (n = 21): sham int. [10-min qd]  EXP (n = 21): laser acupuncture + cupping [10-min qd]  int. duration: 5-d  follow-up time: none  medications: none | VAS  Ryodoraku values | everyday pre- and post-int. |
| Liu, et al. (2011)  [70] | CTR: 51.45 ± 9.28  EXP: 52.35 ± 8.51  / NSLBP | CTR (M = 12, F = 28): inferential current [10×]  EXP (M = 12, F = 28): bioelectricity resonance therapy [5×]  int. duration: 10-d  follow-up time: none  medications: none | VAS  PPI  overall efficacy (symptoms improvement) | pre- and post-int. |
| Vong, et al. (2011)  [72] | CTR: 45.1 ± 10.7  EXP: 44.6 ± 11.2  / CLBP: > 3-mo | CTR (M = 16, F = 22): inferential current [30-min/sess. 10 sess.] + LDE  EXP (M = 12, F = 26): CTR + MET [30-min/sess. 10 sess.]  int. duration: 8-w  follow-up time: 1-mo  medications: none | VAS  RMDQ  trunk ROM  SF-36 | pre- and post-int., 1-mo post-int. |
| Zhu, Li, & Jin (2010)  [73] | CTR: 52.83 ± 11.74  EXP: 53.15 ± 13.62  / LBP: LDH | CTR (M = 17, F = 13): traction [60-min 1×/d]  EXP (M = 18, F = 14): TCE [45-min 2×/d]  int. duration: 2-w  follow-up time: none  medications: none | VAS  RMDQ | pre- and post-int., 3-mo, and 6-mo post-int. |
| Suen & Wong (2008), Suen, et al. (2007)  [74,75] | CTR: 81.23 ± 6.21  EXP: 82.13 ± 6.87  / CNSLBP: > 3-mo | CTR (M = 0, F = 30): auricular taping with semen vaccariae [one ear q3d]  EXP (M = 2, F = 28): auricular taping with magnetic pellets [one ear q3d]  int. duration: 3-w  follow-up time: 4-w  medications: none | NRS  mALBPDS | pre-int., 1.5-w of int., post-int., 2-w, and 4-w post-int. |
| Yip & Tse (2004)  [76] | CTR: 48.1 ± 4.0  EXP: 43.8 ± 3.0  / SANSLBP: < 4-w | CTR (M = 4, F = 25): usual care  EXP (M = 5, F = 27): CTR + intermittent wave EA + acupoint massage [35 – 40-min 8 sess.]  int. duration: 3-w  follow-up time: 1-w  medications: none | VAS  mALBPDS  pain duration  spinal ROM  walking time | pre-int., 1-w post-int. |
| Hsieh & Lee (2002)  [77] | CTR: > 18 yr  EXP1: > 18 yr  EXP2: > 18 yr  / LBP | CTR (M = 10, F = 21): medications [30-min qd]  EXP1 (M = 18, F = 35): CTR + one-shot EA [15-min]  EXP2 (M = 16, F = 33): CTR + one-shot inferential current [15-min]  int. duration: one time  follow-up time: 1-w  medications: diclofenac potassium + mephenoxalone + antacid Wellpine | VAS  QBPDS | pre- and post-int., 3-d, and 1-w post-int. |
