# Supplementary material for: The effectiveness of non-pharmacological interventions for low back pain in China: A systematic review and network meta-analysis
Source: PLoS One. 2025 May 9;20(5):e0322929. doi: 10.1371/journal.pone.0322929 (PMC12063812; doi:10.1371/journal.pone.0322929)
Supplement: S4 Table — (DOCX) [file pone.0322929.s004.docx]

| **Authors & year** | **Randomization process** | **Deviations from intended interventions** | **Missing outcome data** | **Measurement of the outcome** | **Selection of the reported result** | **Overall Bias** |
| --- | --- | --- | --- | --- | --- | --- |
| Gan, et al. (2024) [26] | 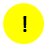 | 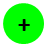 | 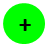 | 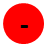 | 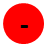 | 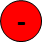 |
| He, et al. (2024) [27] | 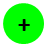 | 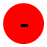 | 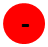 | 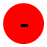 | 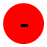 | 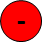 |
| Shi, et al. (2024) [38] | 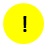 | 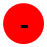 | 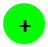 | 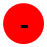 | 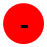 | 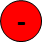 |
| Zheng, et al. (2024) [49] | 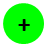 | 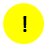 | 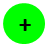 | 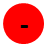 | 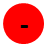 | 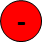 |
| Zuo, et al. (2024) [60] | 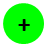 | 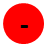 | 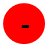 | 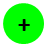 | 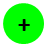 | 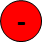 |
| Cheng, et al. (2023) [71] | 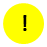 | 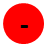 | 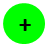 | 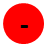 | 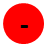 | 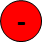 |
| Ju (2023) [78] | 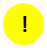 | 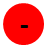 | 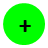 | 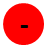 | 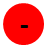 | 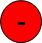 |
| Li, et al. (2023) [79] | 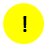 | 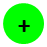 | 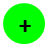 | 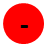 | 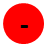 | 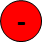 |
| Liu, et al. (2023) [80] | 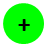 | 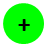 | 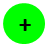 | 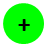 | 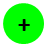 | 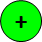 |
| Qiao, et al. (2023) [81] | 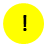 | 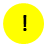 | 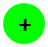 | 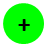 | 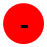 | 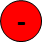 |
| Wang, et al. (2023) [28] | 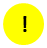 | 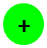 | 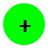 | 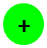 | 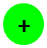 | 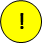 |
| Yang, et al. (2023a) [29] | 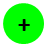 | 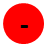 | 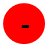 | 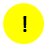 | 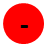 | 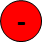 |
| Yang, et al. (2023b) [30] | 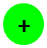 | 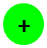 | 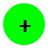 | 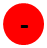 | 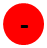 | 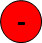 |
| Li (2022) [31] | 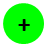 | 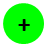 | 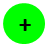 | 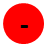 | 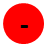 | 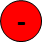 |
| Li, et al. (2022) [32] | 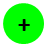 | 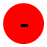 | 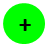 | 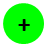 | 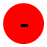 | 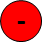 |
| Peng, et al. (2022) [33] | 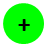 | 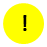 | 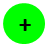 | 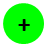 | 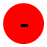 | 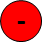 |
| Wang, et al. (2022) [36] | 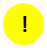 | 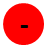 | 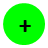 | 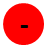 |  |  |
| Chen, et al. (2022) [34] |  |  |  |  |  |  |
| Huo, et al. (2022) [35] |  |  |  |  |  |  |
| Zheng, et al. (2022) [37] |  |  |  |  |  |  |
| Zhou, et al. (2022) [39] |  |  |  |  |  |  |
| Guo, et al. (2021) [40] |  |  |  |  |  |  |
| Hao (2021) [41] |  |  |  |  |  |  |
| Hu, et al. (2021) [42] |  |  |  |  |  |  |
| Li & Zhou (2021) [43] |  |  |  |  |  |  |
| Lu, et al. (2021a) [44] |  |  |  |  |  |  |
| Lu, et al. (2021b) [45] |  |  |  |  |  |  |
| Ma, et al. (2021) [46] |  |  |  |  |  |  |
| Yao, et al. (2020) [47] |  |  |  |  |  |  |
| Yeh, et al. (2020) [48] |  |  |  |  |  |  |
| Hu, et al. (2019) [82] |  |  |  |  |  |  |
| Li, Yan & Huang (2019) [50] |  |  |  |  |  |  |
| Luo, et al. (2019) [51] |  |  |  |  |  |  |
| Zou, et al. (2019) [52] |  |  |  |  |  |  |
| Wang, et al. (2018a) [53] |  |  |  |  |  |  |
| Wang, et al. (2018b) [54] |  |  |  |  |  |  |
| Bi, et al. (2017) [55] |  |  |  |  |  |  |
| Xiao, et al. (2017) [56] |  |  |  |  |  |  |
| Wang, et al. (2017) [57] |  |  |  |  |  |  |
| Tang, et al. (2016) [58] |  |  |  |  |  |  |
| Xu, et al. (2015) [59] |  |  |  |  |  |  |
| Ye, et al. (2015) [61] |  |  |  |  |  |  |
| Zhang, et al. (2015) [62] |  |  |  |  |  |  |
| Bahrami-Taghanaki, et al. (2014) [65] |  |  |  |  |  |  |
| Chen, et al. (2014) [83] |  |  |  |  |  |  |
| Hsieh & Lee (2014) [64] |  |  |  |  |  |  |
| Zhang, et al. (2014) [63] |  |  |  |  |  |  |
| Feng, et al. (2013) [67] |  |  |  |  |  |  |
| Wu, et al. (2013) [66] |  |  |  |  |  |  |
| Kong, et al. (2012) [68] |  |  |  |  |  |  |
| Lin, et al. (2012) [69] |  |  |  |  |  |  |
| Liu, et al. (2011) [70] |  |  |  |  |  |  |
| Vong, et al. (2011) [72] |  |  |  |  |  |  |
| Zhu, et al. (2010) [73] |  |  |  |  |  |  |
| Suen & Wong (2008), Suen, et al. (2007) [74,75] |  |  |  |  |  |  |
| Yip & Tse (2004) [76] |  |  |  |  |  |  |
| Hsieh & Lee (2002) [77] |  |  |  |  |  |  |
