# Supplementary material for: The effectiveness of non-pharmacological interventions for low back pain in China: A systematic review and network meta-analysis
Source: PLoS One. 2025 May 9;20(5):e0322929. doi: 10.1371/journal.pone.0322929 (PMC12063812; doi:10.1371/journal.pone.0322929)
Supplement: S5 Table — (DOCX) [file pone.0322929.s005.docx]

| Pairwise comparison | Imprecision | Heterogeneity |
| --- | --- | --- |
| Pain intensity | | |
| acupuncture:medications | Some concerns | Some concerns |
| cupping and scraping:medications | Some concerns | Some concerns |
| medications:medications+PENS | Some concerns | No concerns |
| medications:medications+TENS | Some concerns | No concerns |
| medications:moxibustion | Some concerns | Major concerns |
| CSE:medications | Some concerns | Some concerns |
| CSE+ESWT:medications | Major concerns | No concerns |
| CSE+PENS:medications | Major concerns | No concerns |
| CSE+TENS:medications | Major concerns | No concerns |
| CSE+manipulation:medications | Major concerns | No concerns |
| CSE+massage:medications | Major concerns | No concerns |
| CSE+mental related:medications | Major concerns | No concerns |
| LDE:medications | Some concerns | Some concerns |
| LDE+mental related:medications | Some concerns | Some concerns |
| medications:PENS | Some concerns | Some concerns |
| medications:PENS minus | Some concerns | Some concerns |
| medications:PENS plus | Major concerns | No concerns |
| medications:TCE | Some concerns | Some concerns |
| medications:TCE+massage | Some concerns | Some concerns |
| medications:TENS | Major concerns | No concerns |
| medications:TENS+thermo | Some concerns | Some concerns |
| medications:TENS+thermo+kinesiotape | Major concerns | No concerns |
| acupuncture plus:medications | Some concerns | Some concerns |
| acupuncture+thermo:medications | Some concerns | No concerns |
| bioelectricity resonance:medications | Major concerns | No concerns |
| massage:medications | Some concerns | No concerns |
| massage+traction:medications | Some concerns | Some concerns |
| medications:medications+CSE | Major concerns | No concerns |
| medications:medications+CSE+ESWT | Major concerns | No concerns |
| medications:moxibustion minus | Some concerns | No concerns |
| medications:moxibustion plus | Some concerns | Some concerns |
| medications:no intervention | No concerns | Some concerns |
| medications:traction | Major concerns | No concerns |
| medications:usual care | Some concerns | No concerns |
| medications:usual care+CSE | Some concerns | Some concerns |
| medications:usual care+LDE | Some concerns | Some concerns |
| medications:usual care+PENS+massage | Some concerns | Some concerns |
| medications:usual care+TCE | Some concerns | Some concerns |
| medications:usual care+medications | Some concerns | Some concerns |
| medications:usual care+medications+acupuncture | Some concerns | Some concerns |
| medications:usual care+medications+manipulation | No concerns | Some concerns |
| Functional disability | | |
| acupuncture:medications | No concerns | Some concerns |
| cupping and scraping:medications | Major concerns | No concerns |
| medications:medications+PENS | Major concerns | No concerns |
| medications:medications+TENS | Major concerns | No concerns |
| medications:moxibustion | No concerns | Some concerns |
| CSE+massage:medications | Some concerns | Some concerns |
| CTE+massage:medications | Some concerns | Some concerns |
| acupuncture plus:medications | No concerns | Some concerns |
| acupuncture+thermo:medications | No concerns | Some concerns |
| massage:medications | Some concerns | Some concerns |
| massage+traction:medications | Some concerns | Some concerns |
| medications:medications+acupuncture | Some concerns | Some concerns |
| medications:moxibustion minus | No concerns | Some concerns |
| medications:moxibustion plus | No concerns | No concerns |
| medications:usual care+medications | Some concerns | Some concerns |
| medications:usual care+medications+acupuncture | Major concerns | No concerns |
| medications:usual care+medications+manipulation | No concerns | Some concerns |
| Overall efficacy | | |
| acupuncture:medications | Major concerns | No concerns |
| cupping and scraping:medications | No concerns | Major concerns |
| medications:moxibustion | Some concerns | Some concerns |
| acupuncture plus:medications | No concerns | Major concerns |
| acupuncture+massage:medications | No concerns | Major concerns |
| acupuncture+moxibustion:medications | Major concerns | No concerns |
| massage+traction:medications | Major concerns | No concerns |
| medications:usual care+medications | Major concerns | No concerns |
