# Supplementary material for: The effectiveness of non-pharmacological interventions for low back pain in China: A systematic review and network meta-analysis
Source: PLoS One. 2025 May 9;20(5):e0322929. doi: 10.1371/journal.pone.0322929 (PMC12063812; doi:10.1371/journal.pone.0322929)
Supplement: S6 Table — (DOCX) [file pone.0322929.s006.docx]

| Gelman-Rubin Diagnostic | | | | | |
| --- | --- | --- | --- | --- | --- |
| Parameter | | Point estimate | | Upper Confidence Interval | |
| d[2] | | 1.004601 | | 1.015801 | |
| d[3] | | 1.004397 | | 1.015320 | |
| d[4] | | 1.000818 | | 1.002571 | |
| d[5] | | 1.009610 | | 1.027893 | |
| d[6] | | 1.015069 | | 1.042510 | |
| d[7] | | 1.011641 | | 1.034402 | |
| d[8] | | 1.010697 | | 1.030551 | |
| d[9] | | 1.005309 | | 1.016175 | |
| d[10] | | 1.010870 | | 1.030262 | |
| d[11] | | 1.011123 | | 1.030833 | |
| d[12] | | 1.011738 | | 1.034710 | |
| d[13] | | 1.013974 | | 1.041514 | |
| d[14] | | 1.005188 | | 1.015506 | |
| d[15] | | 1.000038 | | 1.000115 | |
| d[16] | | 1.012219 | | 1.037021 | |
| d[17] | | 1.010405 | | 1.031727 | |
| d[18] | | 1.006718 | | 1.020236 | |
| d[19] | | 1.000770 | | 1.002343 | |
| d[20] | | 1.010021 | | 1.029590 | |
| d[21] | | 1.009407 | | 1.027698 | |
| d[22] | | 1.000250 | | 1.000812 | |
| d[23] | | 1.000195 | | 1.000408 | |
| d[24] | | 1.002118 | | 1.006774 | |
| d[25] | | 1.001876 | | 1.006189 | |
| d[26] | | 1.001647 | | 1.005669 | |
| d[27] | | 1.010954 | | 1.030554 | |
| d[28] | | 1.012378 | | 1.035887 | |
| d[29] | | 1.011271 | | 1.032659 | |
| d[30] | | 1.010168 | | 1.030259 | |
| d[31] | | 1.011736 | | 1.034407 | |
| d[32] | | 1.009474 | | 1.030170 | |
| d[33] | | 1.007670 | | 1.025386 | |
| d[34] | | 1.010142 | | 1.031136 | |
| d[35] | | 1.013789 | | 1.040930 | |
| d[36] | | 1.011371 | | 1.035097 | |
| d[37] | | 1.011853 | | 1.035567 | |
| d[38] | | 1.011991 | | 1.036203 | |
| d[39] | | 1.002757 | | 1.009388 | |
| d[40] | | 1.002347 | | 1.007739 | |
| d[41] | | 1.001850 | | 1.006484 | |
| d[42] | | 1.011867 | | 1.034407 | |
| sigma | | 1.002384 | | 1.008538 | |
| mpsrf | | 1.011346 | |  | |
| Geweke Diagnostic | | | | | |
| Parameter | Chain 1 | | Chain 2 | | Chain 3 |
| d[2] | 0.8090240 | | -0.631118073 | | -1.39716899 |
| d[3] | 0.7471027 | | -0.462251529 | | -1.39307335 |
| d[4] | 0.6365509 | | -0.576115106 | | -1.08482688 |
| d[5] | 0.5997371 | | -0.378600277 | | -0.25283695 |
| d[6] | 0.3586568 | | -0.367236349 | | -0.20056239 |
| d[7] | 0.2899045 | | -0.253207419 | | -0.39689025 |
| d[8] | 0.4133885 | | -0.366924058 | | -0.14915811 |
| d[9] | 0.4788747 | | -0.474163221 | | -0.50851706 |
| d[10] | 0.4351606 | | -0.274013828 | | -0.24898190 |
| d[11] | 0.3966072 | | -0.299286065 | | -0.15139263 |
| d[12] | 0.4248710 | | -0.307550937 | | -0.13633086 |
| d[13] | 0.3407765 | | -0.399798655 | | -0.15390345 |
| d[14] | 0.5621528 | | -0.454747041 | | -0.49440477 |
| d[15] | 1.2135745 | | -1.138427527 | | -1.53671171 |
| d[16] | 0.2588081 | | -0.367438864 | | 0.09217366 |
| d[17] | 0.3363532 | | -0.439748362 | | 0.32444885 |
| d[18] | 0.5092906 | | -0.466451317 | | -0.44486631 |
| d[19] | 0.5122101 | | -0.148818860 | | -1.01703354 |
| d[20] | 0.2991081 | | -0.258247263 | | -0.47050324 |
| d[21] | 0.2218310 | | -0.398217805 | | -0.55124117 |
| d[22] | 1.2911414 | | 0.098421697 | | -1.73357951 |
| d[23] | 1.5094741 | | -0.368171042 | | -2.07685608 |
| d[24] | 0.7913629 | | -1.002658138 | | -0.93421113 |
| d[25] | 0.4221011 | | -0.831011556 | | -0.88566977 |
| d[26] | 0.6213978 | | -0.989170208 | | -1.44925168 |
| d[27] | 0.4213762 | | -0.421242353 | | -0.30904735 |
| d[28] | 0.6475263 | | -0.275228833 | | -0.28625316 |
| d[29] | 0.8212950 | | -0.000479179 | | -0.09316887 |
| d[30] | 0.7177444 | | -0.084113887 | | -0.25302113 |
| d[31] | 0.5128166 | | -0.306803654 | | -0.20313953 |
| d[32] | 0.1383993 | | -0.222802610 | | 0.10334844 |
| d[33] | 0.1727587 | | -0.254744642 | | 0.24322967 |
| d[34] | 0.3225747 | | -0.289630008 | | -0.26218926 |
| d[35] | 0.4074610 | | -0.462972226 | | -0.01742899 |
| d[36] | 0.4036539 | | -0.513846110 | | 0.01409741 |
| d[37] | 0.4234241 | | -0.462825669 | | -0.05791249 |
| d[38] | 0.4270689 | | -0.488944174 | | -0.01628931 |
| d[39] | 0.6730335 | | -0.534197830 | | -1.30752035 |
| d[40] | 1.0574710 | | -0.565808392 | | -1.67378791 |
| d[41] | 0.1103204 | | -0.403698025 | | -0.35056013 |
| d[42] | 0.3695379 | | -0.473446154 | | -0.06873463 |
| sigma | -0.9006041 | | -0.999236600 | | -1.46777324 |
