# Supplementary material for: The effectiveness of non-pharmacological interventions for low back pain in China: A systematic review and network meta-analysis
Source: PLoS One. 2025 May 9;20(5):e0322929. doi: 10.1371/journal.pone.0322929 (PMC12063812; doi:10.1371/journal.pone.0322929)
Supplement: S9 Table — (DOCX) [file pone.0322929.s009.docx]

| Gelman-Rubin Diagnostic | | | | | |
| --- | --- | --- | --- | --- | --- |
| Parameter | | Point estimate | | Upper Confidence Interval | |
| d[2] | | 1.0026376 | | 1.008344 | |
| d[3] | | 1.0025387 | | 1.008039 | |
| d[4] | | 1.0011126 | | 1.003935 | |
| d[5] | | 1.0018035 | | 1.005859 | |
| d[6] | | 1.0012976 | | 1.004626 | |
| d[7] | | 1.0002217 | | 1.000876 | |
| d[8] | | 1.0023591 | | 1.008197 | |
| d[9] | | 1.0001636 | | 1.000332 | |
| d[10] | | 1.0008933 | | 1.003024 | |
| d[11] | | 1.0000989 | | 1.000473 | |
| d[12] | | 0.9999646 | | 1.000040 | |
| d[13] | | 1.0008270 | | 1.002492 | |
| d[14] | | 1.0011911 | | 1.003468 | |
| d[15] | | 1.0006825 | | 1.002165 | |
| d[16] | | 1.0020388 | | 1.007236 | |
| d[17] | | 1.0018333 | | 1.006444 | |
| d[18] | | 1.0017497 | | 1.006505 | |
| sigma | | 1.0002802 | | 1.000717 | |
| mpsrf | | 1.003176 | |  | |
| Geweke Diagnostic | | | | | |
| Parameter | Chain 1 | | Chain 2 | | Chain 3 |
| d[2] | -0.09496614 | | -0.37190163 | | -2.7106585 |
| d[3] | -0.18298282 | | -0.37108364 | | -2.3645082 |
| d[4] | 0.09005771 | | 0.22525764 | | -2.0519108 |
| d[5] | -0.33083053 | | -0.18147237 | | -2.6156685 |
| d[6] | -0.56099545 | | -0.08403051 | | -2.9206273 |
| d[7] | -0.50368559 | | 0.33939902 | | -0.1350396 |
| d[8] | -0.26786042 | | -0.19505972 | | -2.7802033 |
| d[9] | -0.86824816 | | -0.86145041 | | -2.4388585 |
| d[10] | 0.06227923 | | -0.38650963 | | -2.1044957 |
| d[11] | 1.05025189 | | -0.18041299 | | 0.6657198 |
| d[12] | 0.72095514 | | -0.75666717 | | -0.9248590 |
| d[13] | 0.05958679 | | -0.46132196 | | -2.2784661 |
| d[14] | 0.10185348 | | -0.21584692 | | -2.6062914 |
| d[15] | 0.15564342 | | -0.47466927 | | -2.4179580 |
| d[16] | -0.75920220 | | -0.36451449 | | -2.0900606 |
| d[17] | -0.82018560 | | -0.31093803 | | -1.8145648 |
| d[18] | -1.07636648 | | -0.20289698 | | -2.4030220 |
| sigma | -0.21229770 | | -0.05230002 | | 1.3009307 |
