# Supplementary material for: The effectiveness of non-pharmacological interventions for low back pain in China: A systematic review and network meta-analysis
Source: PLoS One. 2025 May 9;20(5):e0322929. doi: 10.1371/journal.pone.0322929 (PMC12063812; doi:10.1371/journal.pone.0322929)
Supplement: S12 Table — (DOCX) [file pone.0322929.s012.docx]

| Gelman-Rubin Diagnostic | | | | | |
| --- | --- | --- | --- | --- | --- |
| Parameter | | Point estimate | | Upper Confidence Interval | |
| d[2] | | 1.000488 | | 1.000871 | |
| d[3] | | 1.000340 | | 1.000693 | |
| d[4] | | 1.001169 | | 1.001788 | |
| d[5] | | 1.000429 | | 1.000877 | |
| d[6] | | 1.000204 | | 1.000302 | |
| d[7] | | 1.000146 | | 1.000348 | |
| d[8] | | 1.000529 | | 1.000594 | |
| d[9] | | 1.000454 | | 1.001012 | |
| sigma | | 1.000697 | | 1.002677 | |
| mpsrf | | 1.001393 | |  | |
| Geweke Diagnostic | | | | | |
| Parameter | Chain 1 | | Chain 2 | | Chain 3 |
| d[2] | 1.2000472 | | -0.82860590 | | 0.41113919 |
| d[3] | 1.0525788 | | -0.56454528 | | 0.62657043 |
| d[4] | 1.1874316 | | -1.60052545 | | 0.29220158 |
| d[5] | 0.8032276 | | -1.03134926 | | 0.01480608 |
| d[6] | -0.1491661 | | 0.73027034 | | 1.26444883 |
| d[7] | -0.6795188 | | 0.05879542 | | 0.86385587 |
| d[8] | 0.2051286 | | -0.23577144 | | 0.79805240 |
| d[9] | 1.1284786 | | -0.79246549 | | 0.27418388 |
| sigma | -2.0743160 | | -2.06535238 | | 1.01122622 |
